# RIPK3 promotes skin inflammation by enhancing IL-36α signaling and necroptosis in keratinocytes

**DOI:** 10.1038/s41419-025-08096-9

**Published:** 2025-10-24

**Authors:** Qing-qing Li, Tao Yang, Jin-jin Ren, Zhi-zhen Hui, Shu-yue Lei, Chun-lan Feng, Xiao-qian Yang, Wei Tang

**Affiliations:** 1 The Institute of Clinical Pharmacology, Anhui Medical University, Key Laboratory of Anti-inflammatory and Immune Medicine, Ministry of Education, Anhui Collaborative Innovation Center of Anti-inflammatory and Immune Medicine, Hefei, China; 2 State Key Laboratory of Chemical Biology, Shanghai Institute of Materia Medica, Chinese Academy of Sciences, Shanghai, China; 3 School of Pharmacy, University of Chinese Academy of Sciences, Beijing, China; 4 School of Chinese Materia Medica, Nanjing University of Chinese Medicine, Nanjing, China

**Keywords:** Cell death and immune response, Immunological disorders

## Abstract

Psoriasis is a chronic inflammatory skin disease characterized by complex pathogenesis involving multiple factors. Keratinocytes, as key structural components, play a critical role in immune regulation and contribute to disease progression through interactions with various immune cells. Receptor-interacting protein kinase 3 (RIPK3) is well-known for its role in necroptosis, acting alongside RIPK1 and mixed-lineage kinase domain-like (MLKL). While studies have shown that inhibitors of necroptosis could alleviate psoriasis-like skin inflammation, direct genetic evidence of RIPK3 is lacking. Furthermore, recent studies have highlighted RIPK3’s independent biological functions beyond necroptosis, yet its pathological role in inflammatory skin disease remains poorly understood. This study aimed to elucidate the pathological role of RIPK3 in the progression of skin inflammation, particularly in keratinocytes. We demonstrated that RIPK3 expression was significantly upregulated in psoriasis patients and mice with imiquimod (IMQ)-induced skin inflammation. Importantly, keratinocyte-specific knockout of RIPK3 using gene-editing tools significantly alleviated IMQ-induced skin inflammation in mice. Interestingly, the absence of RIPK3 not only inhibited necroptosis and associated inflammatory responses but also significantly reduced interleukin-36α (IL-36α) expression in keratinocytes. IL-36α, known to drive skin inflammation, promote immune cell recruitment, and disrupt the epidermal barrier, is a critical mediator of inflammatory skin disease pathogenesis. Further investigation using MLKL-knockout mice and keratinocytes revealed that RIPK3 regulates the IL-36α/NF-κB signaling axis through an MLKL-independent mechanism. Collectively, our findings uncover a dual pathogenic role for RIPK3 in skin inflammation: promoting inflammation through both canonical necroptosis and a distinct, non-necroptotic pathway that drives IL-36α activation. These insights not only identify RIPK3 as a potential therapeutic target for psoriasis-like skin inflammation but also uncover its previously unappreciated roles in inflammatory diseases beyond necroptosis.

## Introduction

Psoriasis is a prevalent chronic inflammatory skin disorder characterized by keratinocyte (KC) proliferation and differentiation, immune cell infiltration, and excessive release of pro-inflammatory mediators [1–3]. In the pathogenesis of psoriasis, keratinocytes not only constitute a critical structural barrier but also play a pivotal role in immune regulation [4–7]. Through dynamic interactions with dendritic cells, T cells, neutrophils and other immune cells, keratinocytes contribute significantly to the initiation and progression of psoriasis [8–11]. Consequently, investigating the aberrant functions of keratinocytes is essential for elucidating the underlying mechanisms of inflammatory skin diseases and advancing the development of novel therapeutic interventions.

Necroptosis is a form of regulated cell death mediated by the receptor-interacting protein kinase (RIPK) family, particularly RIPK1 and RIPK3 [12]. In the absence or inhibition of apoptotic signaling, death receptors activate the RIPK1/RIPK3 complex, which subsequently phosphorylates mixed-lineage kinase domain-like (MLKL) proteins [13–15]. Phosphorylation of MLKL triggers its oligomerization and translocation to the plasma membrane, where it forms pores that disrupt membrane integrity, culminating in cellular rupture and necroptotic cell death [16, 17]. Necroptosis is closely implicated in the pathogenesis of various inflammatory diseases [18]. Emerging evidence has highlighted a significant association between keratinocyte necroptosis and the development of inflammatory skin disorders [19, 20]. Inhibition of keratinocyte necroptosis has been demonstrated to alleviate imiquimod (IMQ)-induced psoriatic inflammation in mice [21, 22]. Furthermore, the administration of a small-molecule RIPK1 inhibitor has been shown to ameliorate skin inflammation by specifically suppressing necroptosis, thereby reducing the inflammatory response and promoting skin homeostasis [19, 23, 24]. However, in the study by Valerie J. Ludbrook et al. [25], once-daily administration of 960 mg GSK’2982772 in patients with moderate-to-severe plaque psoriasis failed to demonstrate meaningful clinical efficacy, resulting in discontinuation of the trial. Keratinocyte necroptosis and associated skin inflammation in epidermal cell-specific RIPK1 knockout mice can be effectively inhibited by the deletion of RIPK3 or MLKL [26], suggesting that RIPK1 plays a crucial role in maintaining the homeostasis and survival of epidermal cells, highlighting the complexity of its regulatory functions in keratinocyte biology. Additionally, RIPK3 directly exacerbates skin inflammation by modulating the production of chemokines and cytokines in keratinocytes, thereby promoting the recruitment of neutrophils to the site of inflammation [27].

Here, we demonstrate that RIPK3 expression is significantly upregulated in patients with psoriasis and IMQ-induced skin inflammation. Then, it was found that RIPK3 deficiency in keratinocytes attenuated IMQ-induced skin inflammation in mice. In addition, we observed that RIPK3 deficiency significantly inhibited keratinocyte necroptosis and effectively blocked the IL-36α signaling pathway. IL-36α, a key member of the IL-1 family, is predominantly expressed by keratinocytes in the skin [28]. Its overexpression disrupts epidermal barrier function and interacts with keratinocytes and immune cells to promote the release of inflammatory mediators [29, 30]. Clinical studies have demonstrated that IL-36R antagonists effectively inhibit psoriatic lesion formation and significantly attenuate the associated inflammatory response [31, 32]. In our study, RIPK3 knockout suppresses the activation of the IL-36α downstream NF-κB signaling pathway, and this effect is independent of MLKL. Our findings underscore the critical role of RIPK3 in keratinocyte-mediated skin inflammation and highlight its potential as a therapeutic target for inflammatory skin diseases, including psoriasis.

## Results

### RIPK3 is upregulated in patients with psoriasis and IMQ-treated mice

**Fig. 1:**
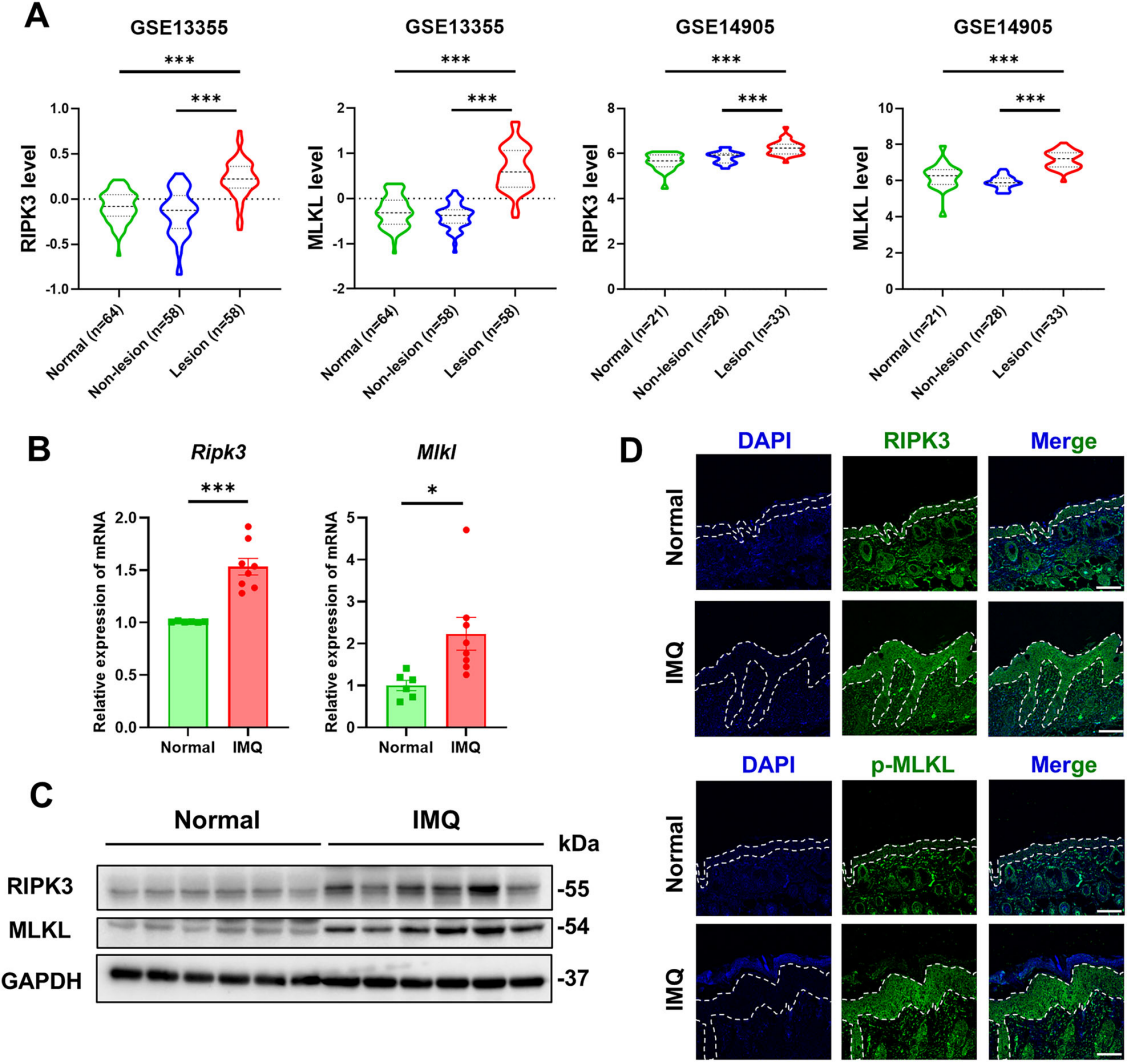
RIPK3 is upregulated in patients with psoriasis and IMQ-treated mice. **A** The mRNA expression levels of *RIPK3* and *MLKL* in the skin from normal, non-lesional skin, and lesional skin of psoriasis patients in the Gene Expression Omnibus (GEO) database (GSE13305 and GSE14905). The mRNA expression levels of *RIPK3* and *MLKL* (**B**) and representative western blot assay of RIPK3 and MLKL (**C**) in the skin of IMQ-treated Balb/c mice. Normal (n = 6), IMQ (n = 8). **D** Immunofluorescence analysis of RIPK3 and p-MLKL in skin tissues of IMQ-treated Balb/c mice. Scale bars, 100 μm. All dates are shown as means ± SEM. ^*^P < 0.05, ^***^P < 0.001, compared as indicated, were measured by one-way ANOVA or Student’s *t*-test.

To investigate the potential role of RIPK3 in psoriasis-like skin inflammation, we queried the open-access Gene Expression Omnibus (GEO) database. This analysis revealed a significant upregulation of *RIPK3* mRNA in psoriatic skin lesions compared to healthy controls (Fig. 1A). Consistent with these findings, further analysis of the GSE13355 and GSE14905 datasets reinforced the elevated *RIPK3* mRNA levels in lesional skin compared to adjacent non-lesional skin (Fig. 1A). Importantly, given that RIPK3 plays a key role in necroptosis through its interaction with MLKL, we observed parallel alterations in *MLKL* expression, underscoring the potential involvement of RIPK3 in psoriasis pathogenesis (Fig. 1A). To further validate these in silico findings, we established a psoriasis-like skin inflammation mouse model through daily topical application of imiquimod (IMQ) to the dorsal skin of Balb/c mice. In lesional skin of these mice, we observed significant increases in both mRNA and protein levels of RIPK3 and MLKL (Fig. 1B, C, and S1). Considering the critical role of keratinocytes in skin inflammation pathogenesis, we performed immunofluorescence staining for RIPK3 and phosphorylated-MLKL (p-MLKL) in skin tissues. Immunofluorescence analysis revealed enhanced expression of both RIPK3 and p-MLKL within keratinocytes of mice with skin inflammation compared to normal animals (Fig. 1D). Collectively, these findings indicate that RIPK3 expression is upregulated in patients with psoriasis and IMQ-treated mice, highlighting its potential role in the pathogenesis of inflammatory skin disease.

### RIPK3^ΔKC^ alleviates the severity of IMQ-induced skin damage

**Fig. 2:**
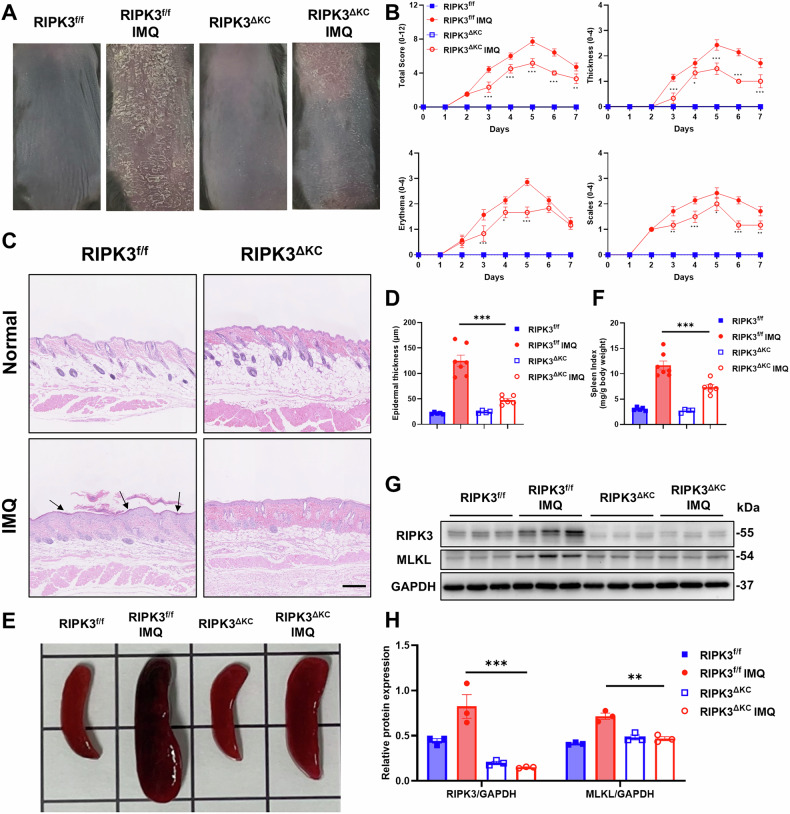
RIPK3^ΔKC^ alleviates the severity of IMQ-induced skin damage. **A** Representative photographs of the back skin in each group (Day 5). **B** Daily thickening, erythema, scaling scores (0-4) and total scores (0-12) in each group. Representative H&E staining images (**C**) and epidermal thickness (**D**) in each group. Scale bars, 250 μm. Representative splenic images (**E**) and splenic index (**F**) in each group. Representative western blot assay (**G**) and quantification (**H**) of RIPK3 and MLKL in the dorsal skin. RIPK3^f/f^ (n = 5), RIPK3^f/f^ IMQ (*n* = 7), RIPK3^ΔKC^ (*n* = 4), RIPK3^ΔKC^ IMQ (*n* = 6). All dates are shown as means ± SEM. ^*^P < 0.05, ^**^P < 0.01, ^***^P < 0.001, compared as indicated, were measured by one-way or two-way ANOVA. There was a significant difference between the RIPK3^f/f^ and RIPK3^f/f^ IMQ groups.

Keratinocytes, the primary epidermal cell type, exhibited marked RIPK3 upregulation in lesional skin, prompting investigation into its epidermal-specific role. To this end, we generated keratinocyte-specific RIPK3 knockout mice (RIPK3^ΔKC^) by crossing RIPK3^f/f^ mice with K14^Cre^ mice. RIPK3^ΔKC^ mice exhibited no overt developmental or gross phenotypic abnormalities compared to Cre-negative littermates, with qPCR confirming significant RIPK3 mRNA reduction in keratinocytes (Fig. S2A-D). In IMQ-induced skin inflammation models, RIPK3^ΔKC^ mice displayed markedly attenuated clinical severity, including reduced scaling, erythema, and epidermal thickening compared to RIPK3^f/f^ mice (Fig. 2A). This improvement was further supported by significantly lower Psoriasis Area Severity Index (PASI) scores in the RIPK3^ΔKC^ mice (Fig. 2B). Munro’s microabscesses is a typical histologic feature of early pathological changes in psoriasis, characterized by abnormal aggregation of neutrophils in the stratum corneum to form tiny abscess-like structures [33, 34]. Histological analysis of skin tissue revealed attenuation of epidermal thickening and a reduction in Munro’s microabscesses in RIPK3^ΔKC^ mice with skin inflammation, as indicated (black arrows, Fig. 2C, D and S2E). Splenomegaly, a hallmark of systemic inflammation, was significantly reduced in IMQ-treated RIPK3^ΔKC^ mice, as reflected by lower spleen weights and splenic index (Fig. 2E, F). Additional protein analyses confirmed that RIPK3^ΔKC^ inhibited the IMQ-induced upregulation of both RIPK3 and MLKL proteins in skin tissues (Fig. 2G, H). Collectively, these data demonstrate that keratinocyte-intrinsic RIPK3 drives skin inflammatory pathology, and its ablation mitigates disease severity.

### RIPK3^ΔKC^ mitigates skin inflammation in IMQ-treated mice

**Fig. 3:**
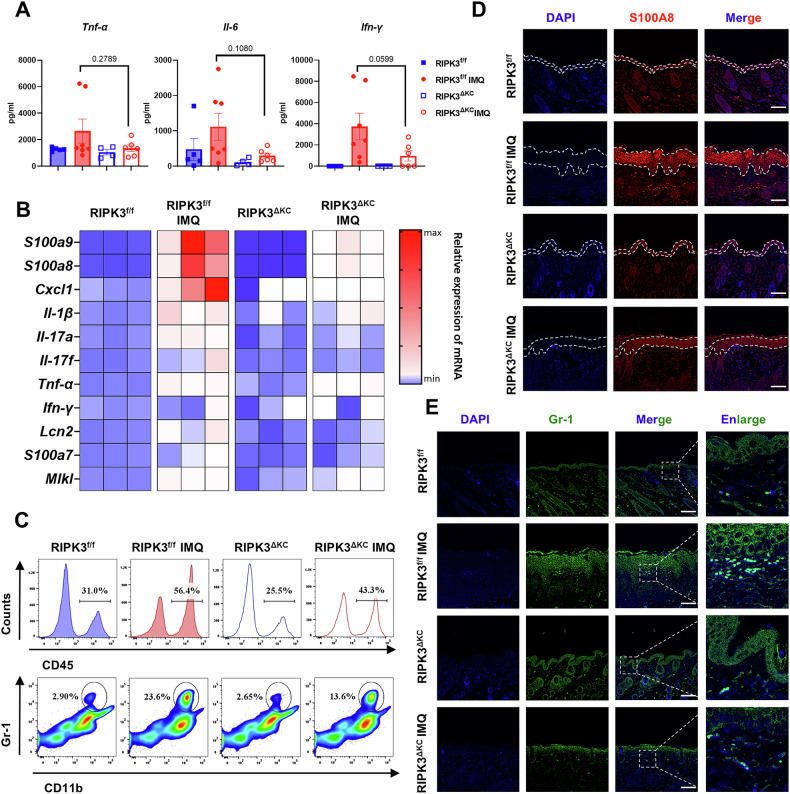
RIPK3^ΔKC^ mitigates skin inflammation in IMQ-treated mice. **A** The level of cytokines (TNF-α, IL-6 and IFN-γ) in serum was measured by ELISA. **B** Heatmap showing mRNA expression levels of cytokines, chemokines and antimicrobial peptides in the skin as measured by RT-PCR. **C** Percentage of leukocytes (CD45^+^) and neutrophils (CD11b^+^Gr-1^+^) cells analyzed by flow cytometry in the skin. Immunofluorescence detection of protein levels of S100A8 (**D**) and Gr-1 (**E**) in the skin. Scale bars, 100 μm. RIPK3^f/f^ (*n* = 5), RIPK3^f/f^ IMQ (*n* = 7), RIPK3^ΔKC^ (*n* = 4), RIPK3^ΔKC^ IMQ (*n* = 6). These results are representative of three independent experiments. All dates are shown as means ± SEM, compared as indicated, were measured by one-way ANOVA.

IMQ induces the release of pro-inflammatory factors and immune cell infiltration through activation of the inflammatory signaling pathways downstream of TLR7, leading to disruption of the inflammatory microenvironment [35]. To define RIPK3’s role in skin inflammation, we analyzed serum cytokines via ELISA and observed reduced levels of TNF-α, IL-6, and IFN-γ in RIPK3^ΔKC^ mice compared to RIPK3^f/f^ mice following IMQ challenge (Fig. 3A). These cytokines are known to be secreted by a variety of immune cells, including activated macrophages, dendritic cells, and T cells, highlighting a broad impact on the inflammatory milieu. Transcriptional profiling of lesional skin further revealed diminished expression of pro-inflammatory mediators (*S100a8*, *S100a9*, *Cxcl1*, *Il-1β*, and *Il-17*) in RIPK3^ΔKC^ mice (Fig. 3B). Notably, *S100a8* and *S100a9* are well-established markers of neutrophil and macrophage activation, and their reduced expression suggests a decrease in the infiltration and activation of these cell types. Furthermore, *Cxcl1* is a potent chemokine primarily involved in neutrophil recruitment, while *Il-1β* and *Il-17* are key cytokines produced by activated macrophages and T cells (particularly Th17 cells), respectively. Flow cytometry of dorsal skin demonstrated that RIPK3^ΔKC^ mice exhibited reduced IMQ-induced leukocyte (CD45^+^) and neutrophil (CD11b^+^Gr-1^+^) infiltration compared to RIPK3^f/f^ mice (Fig. 3C). IMQ-induced skin injury triggers the release of numerous damage-associated molecular patterns (DAMPs), which activate inflammatory pathways such as TLR4 signaling. Among these, S100A8, which is one of the most abundant DAMPs and is highly expressed in lesional skin, amplifies inflammation through interaction with TLR4 and TLR7 [36, 37]. Accordingly, we assessed S100A8 expression in the skin of RIPK3^ΔKC^ mice with skin inflammation. Immunofluorescence analysis corroborated these findings, showing that keratinocyte-specific RIPK3 deletion suppressed S100A8 overexpression in the epidermis (Fig. 3D). Neutrophil aggregation, a hallmark of skin inflammation [38], was markedly attenuated in RIPK3^ΔKC^ mice, with fewer neutrophils localized to dermal lesions compared to RIPK3^f/f^ mice (Fig. 3E). Overall, these findings demonstrate that keratinocyte-specific RIPK3 deletion significantly mitigates the broad inflammatory response in skin by reducing pro-inflammatory cytokine and mediator expression, suppressing neutrophil infiltration and attenuating DAMPs like S100A8.

### Targeting RIPK3 suppresses TSZ-induced keratinocyte necroptosis and inflammation

**Fig. 4:**
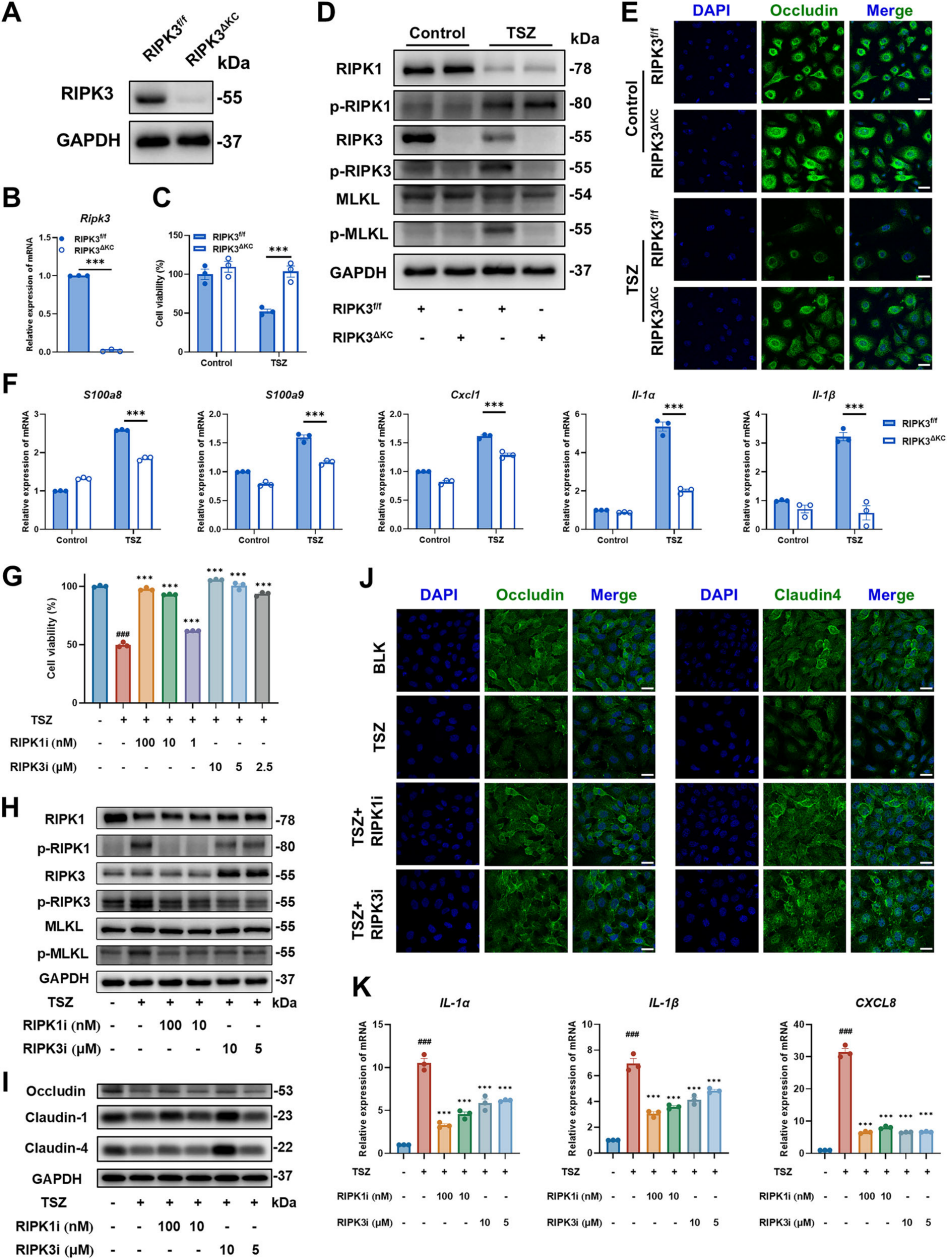
Targeting RIPK3 suppresses TSZ-induced keratinocyte necroptosis and inflammation. Western blot (**A**) and RT-PCR (**B**) analysis of the expression level of RIPK3 in primary keratinocytes from RIPK3^f/f^ and RIPK3^ΔKC^ mice. **C** Cell viability of RIPK3-deficient primary keratinocytes following TSZ treatment was detected by CCK-8 kit. **D** Representative western blot analysis of the necroptosis pathway after TSZ treatment for 12 hours in primary keratinocytes. **E** Representative immunofluorescence analysis of Occludin in primary keratinocytes after TSZ treatment for 6 hours in primary keratinocytes. Scale bars, 100 μm. **F** RT-PCR analysis of mRNA levels of chemokines, cytokines and antimicrobial peptides after TSZ treatment for 6 hours in primary keratinocytes. **G** Cell viability of HaCaT cells treated with GSK’2982772 (100, 10 and 1 nM) and GSK’872 (10, 5 and 2.5 μM) was detected by CellTiter kit. Representative western blot analysis of the necroptosis pathway (**H**) in HaCaT cells treated with TSZ for 24 hours, and tight junction proteins (**I**) in HaCaT cells treated with TSZ for 6 hours. Representative immunofluorescence (**J**) and mRNA expression levels of *IL-1α*, *IL-1β* and *CXCL8* measured by RT-PCR (**K**) in HaCaT cells treated with TSZ for 6 hours. Scale bars, 100 μm. These results are representative of three independent experiments. All dates are shown as means ± SEM. ^***^P < 0.001, compared as indicated, ^###^*P* < 0.001, compared with BLANK, were measured by Student’s *t*-test, one-way or two-way ANOVA. There was a significant difference between the RIPK3^f/f^ and RIPK3^f/f^ TSZ groups.

Pharmacological inhibition of the RIPK1/RIPK3/MLKL axis ameliorates psoriatic inflammation by blocking keratinocyte necroptosis [21–23]. Based on our finding that keratinocyte-specific RIPK3 deletion protects against skin damage in vivo, we isolated primary keratinocytes from neonatal RIPK3^ΔKC^ and RIPK3^f/f^ mice (Fig. 4A, B) and induced necroptosis using the canonical TNF-α/SM164/z-VAD (TSZ) system [39]. Our findings demonstrated that RIPK3 knockout effectively inhibits keratinocyte necroptosis and, specifically, the phosphorylation of RIPK3 and subsequent MLKL activation, thereby confirming its critical involvement in necroptotic signaling (Fig. 4C, D, S3A). The skin barrier is a critical defense mechanism against external pathogens and plays a vital role in maintaining immune homeostasis. Our findings show that RIPK3 deletion significantly ameliorates the reduced expression of tight junction protein Occludin in keratinocytes (Fig. 4E). Similarly, in the IMQ-induced skin inflammation models, toluidine blue staining also showed that RIPK3 deletion protected the epidermal barrier (Fig. S3B). Furthermore, RIPK3 deficiency significantly attenuated the downregulation of chemokine and cytokine gene expression in keratinocytes (Fig. 4F).

Structurally, RIPK3 contains a kinase domain that mediates necroptotic signaling and a RHIM domain [40, 41]. To dissect kinase-specific contributions, we treated TSZ-stimulated HaCaT cells with the RIPK3 inhibitor GSK’872 or the RIPK1 inhibitor GSK’2982772. The RIPK1 and RIPK3 inhibitors effectively reduced HaCaT cell death and inhibited phosphorylation of both RIPK3 and MLKL (Fig. 4G, H and S3C). Moreover, GSK’872 rescued TSZ-induced loss of tight junction proteins (Occludin, Claudin4) in HaCaT cells (Fig. 4I, J and S3D), while also dampening expression of inflammatory mediators (*IL-1α*, *IL-1β*, *CXCL8*) (Fig. 4K). Collectively, these in vitro findings indicate that both genetic ablation and pharmacological inhibition of RIPK3 kinase activity effectively suppress necroptosis and inflammatory responses in keratinocytes.

### RIPK3 amplifies IL-36 signaling in skin inflammation

**Fig. 5:**
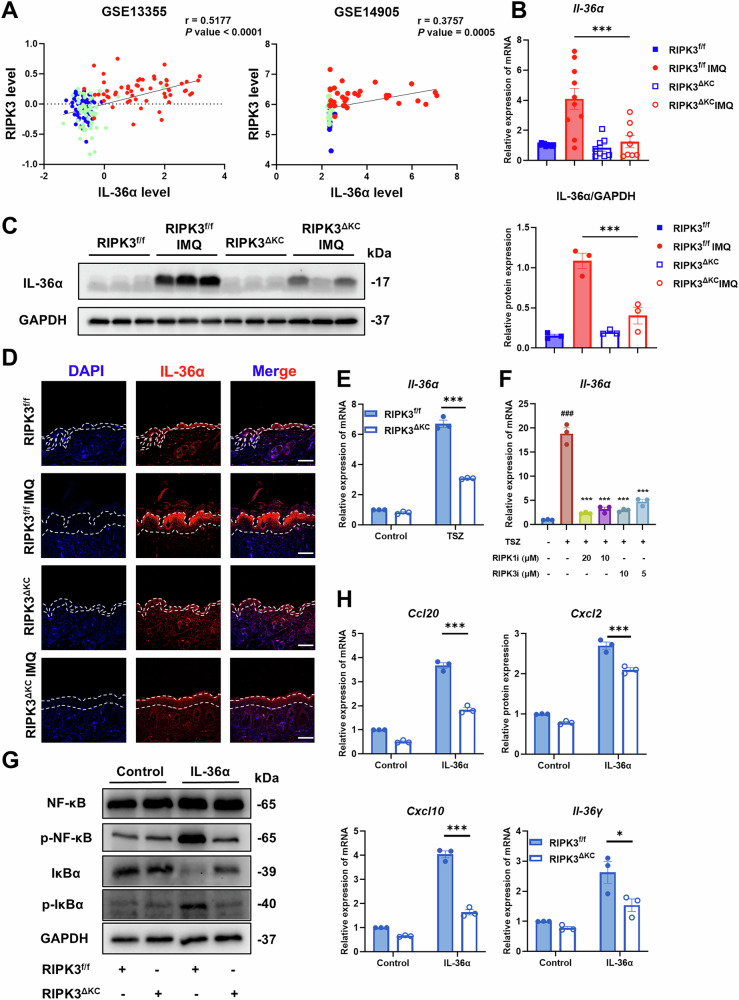
RIPK3 amplifies IL-36 signaling in skin inflammation. **A** Correlation between *IL-36α* and *RIPK3* expression in psoriasis patients in the GEO database. GSE13305: normal (n = 64, green), non-lesional skin (n = 58, blue) and lesional skin (n = 58, red); GSE14905: normal (n = 21, green), non-lesional skin (n = 28, blue) and lesional skin (n = 33, red). The mRNA expression of *Il-36α* (**B**); representative western blot (**C**) analysis of IL-36α protein levels and immunofluorescence staining (**D**) of IL-36α levels in the skin. Scale bars, 100 μm. The mRNA expression of IL-36α in primary keratinocytes from RIPK3^f/f^ and RIPK3^ΔKC^ mice (**E**) and normal mice (**F**) treated with TSZ for 6 hours. **G** Representative western blot assay of IL-36α pathway in primary keratinocytes treated with IL-36α for 15 minutes. **H** The mRNA expression of *Ccl20*, *Cxcl2*, *Cxcl10* and *Il-36γ* in primary keratinocytes treated with IL-36α for 6 hours. These results are representative of three independent experiments. All dates are shown as means ± SEM. ^*^P < 0.05, ^***^P < 0.001, compared as indicated, ^###^*P* < 0.001, compared with BLANK, were measured by one-way or two-way ANOVA. Significant differences were observed between the RIPK3^f/f^ and RIPK3^f/f^ IMQ groups, the RIPK3^f/f^ and RIPK3^f/f^ TSZ groups, and the RIPK3^f/f^ and RIPK3^f/f^ IL-36α groups.

Intercellular communication between immune cells and keratinocytes, mediated by cytokines and their receptors, is a central driver of skin inflammation pathogenesis in diseases like psoriasis [11]. Indeed, certain cytokines such as TNF-α, IL-23/IL-17A axis, and IL-36 have emerged as key mediators in psoriasis, as evidenced by the clinical efficacy of therapies targeting these factors [42, 43]. Our present findings demonstrated that keratinocyte-specific RIPK3 deletion ameliorates IMQ-induced skin damage and cutaneous inflammation in mice, while RIPK3 ablation or kinase inhibition suppresses necroptosis and inflammatory responses in keratinocytes. However, the molecular mechanism linking RIPK3 to skin inflammation remained unclear. To explore the potential involvement of RIPK3 in clinically relevant cytokine pathways, we performed an integrated re-analysis of the GSE13355 and GSE14905 datasets, focusing on the expression of RIPK3 alongside key psoriasis-associated cytokines. Intriguingly, these analyses revealed a positive correlation between *RIPK3* levels and the level of *IL-36α* and *TNF-α* in human psoriatic lesions, while no such significant correlation was observed with *IL-17A* or *IL-23* (Fig. 5A, S4A). Given that RIPK3 is recognized as a downstream signaling component of TNF-α signaling, its potential functional link to IL-36α prompted us to focus our subsequent investigations on IL-36α. Additionally, we analyzed other datasets to confirm the correlation between *IL-36α* and *RIPK3*, finding a similarly significant positive correlation (Fig. S4B). In IMQ-induced skin inflammatory mice, RIPK3^ΔKC^ keratinocytes exhibited marked downregulation of IL-36α mRNA and protein (Fig. 5B–D). Consistent with these in vivo findings, both genetic ablation of RIPK3 and pharmacological treatment with RIPK1/3 inhibitor significantly reduced *Il-36α* mRNA expression in primary keratinocytes (Fig. 5E, F).

These cumulative results strongly suggest that RIPK3 inhibition diminishes IL-36α expression in keratinocytes and likely participates in skin inflammation pathogenesis via modulation of IL-36 signaling within these cells. To directly interrogate this mechanistic hypothesis, we stimulated primary keratinocytes with recombinant IL-36α. IL-36α stimulation induced IκBα and NF-κB phosphorylation in RIPK3^f/f^ keratinocytes, while RIPK3 deficiency blunted this response (Fig. 5G, S5). RIPK3^ΔKC^ keratinocytes also failed to upregulate IL-36α-dependent chemokines (*Ccl20*, *Cxcl2*, *Cxcl10*) and the auto-amplifying cytokine *Il-36γ* (Fig. 5H). Thus, RIPK3 acts as a keratinocyte-specific amplifier of IL-36α signaling, orchestrating a feed-forward loop that sustains cutaneous skin inflammation.

### RIPK3 promotes IL-36α signaling in a MLKL-independent manner in keratinocytes

**Fig. 6:**
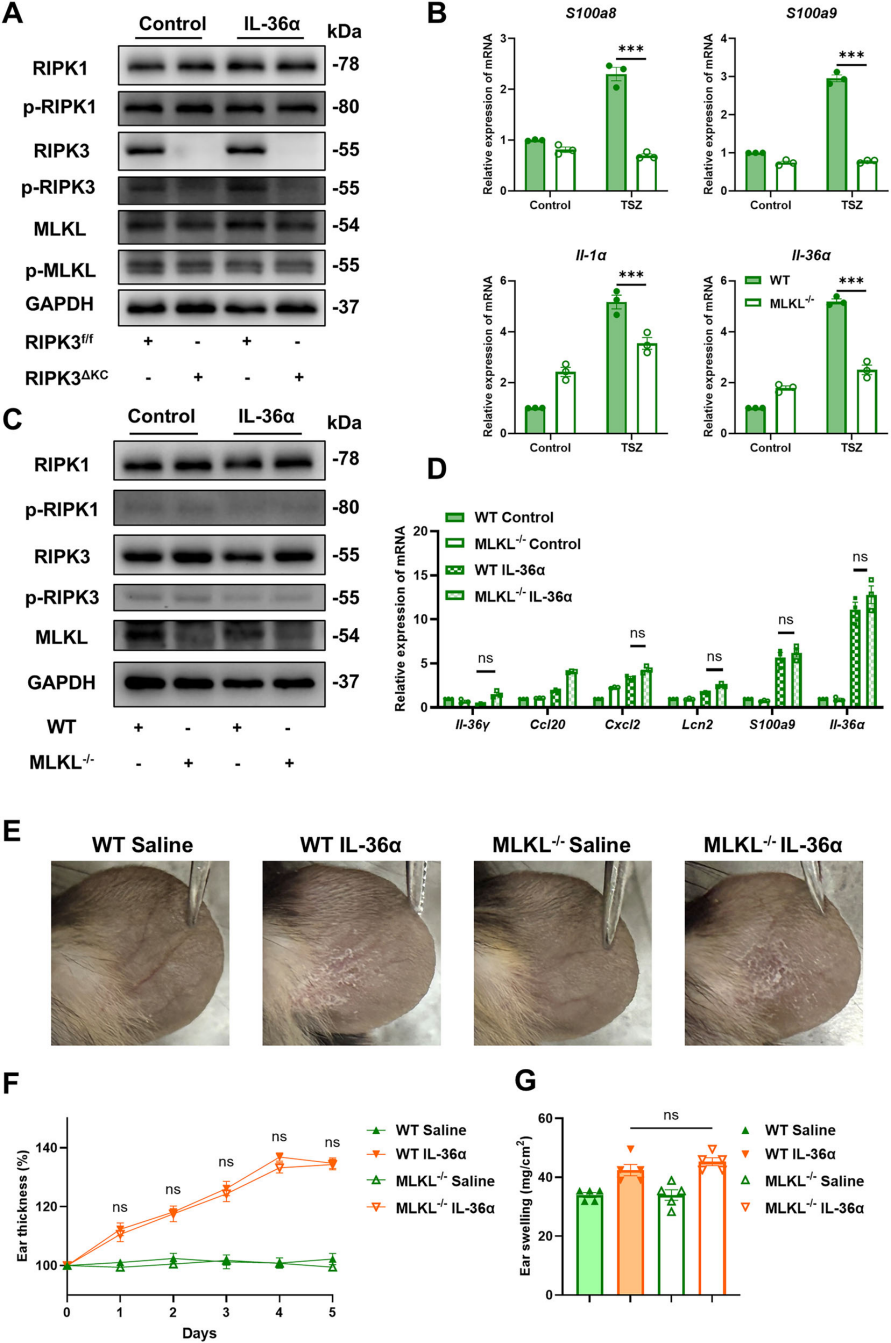
RIPK3 promotes IL-36α signaling in a MLKL-independent manner in keratinocytes. **A** Representative western blot assay of necroptotic proteins in primary keratinocytes from RIPK3^f/f^ and RIPK3^ΔKC^ mice treated with IL-36α for 6 hours. **B** RT-PCR detection of mRNA expression levels of *S100a8*, *S100a9*, *Il-1α* and *Il-36α* after TSZ induction in primary keratinocytes. **C** Representative western blot images assay of necroptotic proteins in IL-36α-induced primary keratinocytes. **D** RT-PCR detection of mRNA expression levels of cytokines such as *Cxcl2*, *Lcn2*, *S100a9* and *Il-36α* after IL-36α induction in primary keratinocytes. **E** Representative images of mice treated with saline and IL-36α at day 5 to demonstrate the scaling of the treated area. Ear thickness (**F**) and ear swelling (**G**) in mice treated with saline and IL-36α in each group. *n* = 5 mice per group. These results are representative of three independent experiments. All dates are shown as means ± SEM. ^***^P < 0.001, ns indicates no significance, compared as indicated, were measured by two-way ANOVA. Significant differences were observed between the WT and WT TSZ groups, as well as between the WT and WT IL-36α groups.

The canonical role of RIPK3 as a central regulator of necroptosis within programmed cell death pathways is well-established [44]. However, recent studies have also highlighted its significant involvement in the regulation of inflammatory responses and apoptosis. In our study, RIPK3 deficiency attenuated IMQ-induced skin damage by suppressing necroptosis and IL-36α-driven inflammation. To dissect whether RIPK3 regulates IL-36α signaling through canonical necroptotic pathways, we first interrogated crosstalk between IL-36α and RIPK1/RIPK3/MLKL. IL-36α stimulation failed to induce phosphorylation of RIPK1, RIPK3, or MLKL in keratinocytes, suggesting RIPK3 modulates IL-36α responses independently of its necroptotic effector MLKL (Fig. 6A, S6A). MLKL, the terminal executor of necroptosis, remains poorly characterized in keratinocyte biology. To define its role in IL-36α signaling, we isolated primary keratinocytes from MLKL^-/-^ mice, confirming MLKL ablation at transcriptional and protein levels (Figs. S6B and 6C). Notably, MLKL deficiency reduced baseline expression of *S100a8*, *S100a9*, and *Il-1a*, reflecting attenuated necroptosis-driven inflammation (Fig. 6B). While MLKL deletion can completely abolish necroptosis, it did not impair IL-36α’s ability to upregulate pro-inflammatory mediators (Fig. 6C, D and S6C–E). To further investigate MLKL’s independence from IL-36α signaling activation, we developed an in vivo model of IL-36α-induced skin inflammation. While IL-36α administration consistently induced ear swelling and desquamation, the genetic ablation of MLKL failed to ameliorate the resulting pathological lesions compared to WT controls (Fig. 6E). Furthermore, MLKL^-/-^ mice exhibited no significant reduction in ear thickening or ear weight relative to the WT group (Fig. 6F, G). These data show that IL-36α drives skin inflammation independently of MLKL. Collectively, our studies reveal a non-necroptotic role for RIPK3 in promoting IL-36α-mediated inflammation, entirely separate from its canonical function with MLKL. This MLKL-independent pathway enables RIPK3 to amplify IL-36α’s pro-inflammatory cascade in keratinocytes, positioning RIPK3 as a multifunctional kinase that integrates necroptotic and cytokine signaling in skin inflammation pathogenesis.

## Discussion

The development of psoriasis is closely linked to the disruption of epidermal homeostasis, with abnormal keratinocyte proliferation and impaired differentiation serving as central pathological features [45]. Beyond serving as the primary effectors responsible for clinical manifestations such as erythema, scaling, and epidermal hyperplasia, keratinocytes also actively modulate the innate immune response in psoriatic inflammation by secreting cytokines, such as chemokines that recruit neutrophils, antimicrobial peptides, and TNF-α [46, 47]. Thus, keratinocytes serve as a critical nexus that bridges genetic susceptibility, immune dysregulation, and metabolic abnormalities in the pathogenesis of the disease.

Excessive proliferation of keratinocytes in skin inflammation is closely associated with dysregulated cell death processes. Recent studies have underscored the pivotal role of aberrant cell death patterns, particularly necroptosis, in the progression of skin inflammation [21, 48–50]. Necroptosis is a highly regulated form of cell death that has significant pathophysiological implications in various inflammatory diseases. The roles of RIPK3 and MLKL in necroptosis have been extensively documented [13]. However, emerging evidence indicates that RIPK3 functions extend beyond its conventional role in MLKL-dependent necroptosis, contributing to tissue damage and modulating inflammatory responses through additional mechanisms [51]. In our study, we generated transgenic mice with specific knockout of RIPK3 in keratinocytes and observed that RIPK3^ΔKC^ significantly ameliorated skin lesions and improved skin inflammation symptoms in mice with IMQ-induced skin inflammation. Our in vitro experiments using primary keratinocytes and HaCaT cells confirmed that RIPK3 intervention markedly inhibited necroptotic signaling. Additionally, RIPK3 intervention not only preserved the barrier function of keratinocytes but also attenuated pro-inflammatory responses.

Necroptosis is characterized by the release of DAMPs, including members of the interleukin-1 family, which intensify inflammatory responses [52]. Both RIPK3 and MLKL have been implicated in the maturation and secretion of IL-1β via activation of the NLRP3 inflammasome. Moreover, RIPK3 could augment inflammatory signaling independently of MLKL by directly modulating IL-1β secretion [53]. Similarly, downregulation of IL-1R1 has been shown to mitigate necroptosis and reduce inflammation in models of cerebral ischemia [54]. IL-36α, a member of the IL-1 family, initiates downstream inflammatory signaling by binding to its receptors, IL-36R and IL-1R AcP, thereby activating pro-inflammatory pathways [28]. In clinical treatment, biologic agents, including TNF-α inhibitors, IL-12/23 inhibitors, IL-17 and its receptor inhibitors, JAK inhibitors, and IL-36R monoclonal antibodies, have been introduced as novel therapeutic strategies, offering new options for psoriasis management [32, 43, 55]. Therefore, in the context of keratinocyte necroptosis, inhibition of RIPK3 may suppress IL-36α production. Our findings demonstrate that both genetic deletion and pharmacological inhibition of RIPK3 lead to a reduction in IL-36α expression, thereby curtailing the self-amplifying inflammatory cascade that typifies skin inflammation. As IL-36α further induces the production of pro-inflammatory cytokines and chemokines, its suppression by RIPK3 inhibition represents a critical intervention point in the disease process [11, 56]. Although the canonical RIPK3-MLKL necroptotic pathway is well established, the data presented here also highlight RIPK3’s involvement in inflammatory regulation beyond MLKL [14, 16]. This observation is consistent with emerging evidence of RIPK3’s role in various inflammatory diseases, including liver disease, acute kidney injury, and inflammatory bowel disease [57–61]. In these settings, RIPK3 modulates additional signaling pathways such as NF-κB, mitochondrial function [62], and inflammasome activation [63].

To further dissect the mechanisms underlying these observations, we developed an MLKL knockdown keratinocyte model. While MLKL deletion effectively reduced necrosis, it did not significantly alter IL-36α-induced inflammatory responses. This finding suggests that RIPK3 may modulate inflammation through MLKL-independent pathways by using MLKL^-/-^ keratinocytes. While our findings in murine models are compelling, we acknowledge their inherent limitations. Future studies will be crucial to validate these RIPK3- and MLKL-independent signaling mechanisms directly in human psoriatic tissue. Such work would definitively confirm the clinical relevance of this pathway and represents an important future direction for our research.

A key finding of this study is that RIPK3 promotes IL-36-induced inflammation independent of its canonical kinase-driven, necroptotic function. This raises the question of how RIPK3 mediates this effect. RIPK3, in this context, might function as a molecular scaffold to potentiate the NF-κB signaling cascade. This hypothesis is supported by existing literature which has documented a physical interaction between RIPK3 and TRAF2, a classic adaptor protein essential for NF-κB activation in other signaling pathways, such as that of TNFR1 [64]. Thus, a similar mechanism is very likely shared in the IL-36 pathway. Downstream of the IL-36 receptor, the E3 ubiquitin ligase TRAF6, a functional analog of TRAF2, is the critical adaptor protein essential for NF-κB activation [65, 66]. Therefore, it is reasonable that RIPK3 may be recruited to the signaling platform via an interaction with the TRAF6-centric complex. While the precise molecular interactions require further experimental investigation, the deduced molecular mechanism provides a plausible explanation for the necroptosis-independent pro-inflammatory function of RIPK3.

**Fig. 7:**
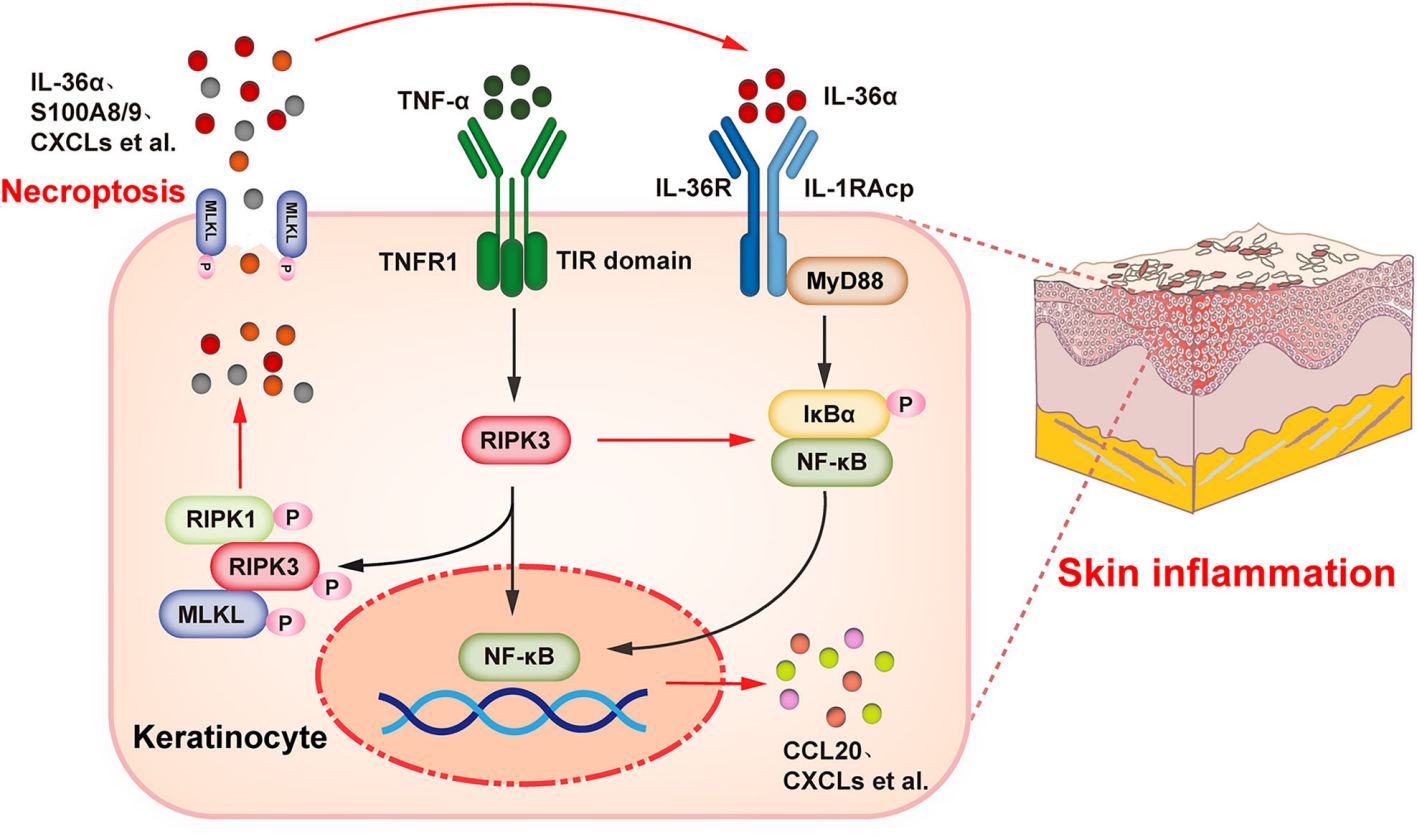
RIPK3 promotes skin inflammation by enhancing IL-36α signaling and necroptosis in keratinocytes. In keratinocytes, RIPK3 orchestrates skin inflammation through a dual pathogenic mechanism. It initiates MLKL-dependent necroptosis, releasing DAMPs like IL-36α. This extracellular IL-36α, in turn, activates the pro-inflammatory NF-κB signaling axis. Beyond merely initiating this cascade, RIPK3 critically functions in an MLKL-independent manner to directly potentiate NF-κB activation, driving robust expression of inflammatory mediators (e.g., CCL20, CXCLs). This interplay constitutes a powerful feed-forward loop that amplifies and sustains cutaneous inflammation.

In summary, our findings demonstrate that RIPK3 not only mediates necroptosis in keratinocytes, thereby exacerbating skin tissue damage, but also amplifies inflammatory responses via the IL-36α signaling pathway. Importantly, in the context of skin inflammation, the regulatory role of RIPK3 on IL-36α-induced inflammation appears to be independent of the classical RIPK3-MLKL axis and may have potential implications for the understanding or treatment of psoriasis (Fig. 7).

## Materials and methods

### Animals

Balb/c mice (6–8 weeks) were obtained from Shanghai Laboratory Animal Center of the Chinese Academy of Sciences. RIPK3^ΔKC^ (RIPK3^f/f^; K14^Cre^) mice were obtained by Cre-LoxP technology, mediated deletion of the RIPK3 gene in exon 4 and exon 9, kindly generated from Zhang’s laboratory. K14^Cre^ mice were purchased from Cyagen (Cyagen CA, USA). MLKL^-/-^ mice were generously provided by Xie’s laboratory. All transgenic mice were created using the C57BL/6 strain as the genetic background. Animals were housed in a specific pathogen-free (SPF) facility of Shanghai Institute of Materia Medica. All experiments were approved by the Institutional Animal Care and Use Committee of the Shanghai Institute of Materia Medica and followed the guidelines set by the Association Assessment and Accreditation of Laboratory Animals Care International. The genotypes of newborn mice were identified using tail-tip PCR, with primer sequences detailed in Table S1.

### Toluidine blue staining

To evaluate epidermal barrier integrity in the IMQ-induced RIPK3^ΔKC^ skin inflammation model, toluidine blue staining was performed as previously described [67]. Dorsal skin tissues were excised using a 7 mm-diameter electric ear punch (Shandong, China), rinsed in PBS, and incubated in 0.1% toluidine blue for 5 minutes at room temperature. Excess dye was removed through extensive PBS washes, and images were captured to document tissue permeability.

### IMQ-induced skin inflammation in mice

Balb/c mice aged 6-8 weeks were shaved on the back and treated with depilatory cream (Veet, Beijing, China), and then randomly divided into two groups: Normal and IMQ. Transgenic mice at 6–8 weeks of age were shaved on their backs and treated with depilatory cream (Veet, Beijing, China) and randomized into four groups: RIPK3^f/f^ group, RIPK3^f/f^ IMQ group, RIPK3^ΔKC^ group and RIPK3^ΔKC^ IMQ group. Mice were treated daily for seven days with 62.5 mg of 5% imiquimod cream, IMQ cream from Sichuan Mingxin Pharmaceutical (Sichuan, China). Clinical lesional area and severity index were assessed daily in all animals during the treatment period [8, 35]. Erythema and scaling were scored independently from 0 to 4 as follows: 0, no abnormality, 1, mild, 2, moderate, 3, moderately severe, and 4, severe. Lesional skin thickening in mice was evaluated macroscopically using a 0–4 scale. Score 0 indicated no thickening, with the skin appearing smooth, without palpable elevation or skin folds. Score 1 denoted slight thickening, characterized by a subtle palpable elevation or minimal skin folding. For moderate thickening (Score 2), clear and obvious skin elevation and folds were visible and palpable, and the skin felt firmer than normal. Severe thickening (Score 3) was defined by skin that was very thick and firm/hard to the touch, with prominent and deep skin folds. Finally, very severe or extreme thickening (Score 4) described skin that was extremely thick, hard, and rigid, potentially accompanied by surface cracking, fissures, or marked deformation of the skin surface.

### IL-36α-induced skin inflammation in mice

Transgenic mice (6–8 weeks old) were randomly assigned to four experimental groups: WT Saline, WT IL-36α, MLKL^-/-^ Saline, and MLKL^-/-^ IL-36α. Mice received daily intradermal injections into the ear for 5 consecutive days, consisting of either 1 μg of murine IL-36α protein (MedChemExpress) or an equivalent volume of saline, as previously described by Anne Müller et al. [68]. Ear thickness was monitored daily using digital Vernier calipers. On day 6, mice were euthanized, and the injected ear tissue was excised using an electric ear punch for gravimetric analysis of tissue edema.

### Skin histopathological examination

Skin tissues from each group were collected, fixed in 10% formalin, and submitted to Servicebio Company (Wuhan, China) for hematoxylin and eosin (H&E) staining. Following staining, skin tissue sections were scanned using a NanoZoomer 2.0 HT digital scanner (Tokyo, Japan), and the epidermal layer thickness was quantitatively analyzed using Image-Pro Plus software (Silver Springs, MD, USA).

### Isolation of mouse primary keratinocytes

Isolation of mouse primary keratinocytes was performed as described previously [69]. The whole skin of neonatal 1 ~ 2-day-old mice was placed in 2 mg/ml of Dispase II (SIGMA, St. Louis, MO, USA) digestion solution at 4 °C overnight. The next day, after 12 hours of digestion in Dispase II, the epidermis and dermis were separated, and the epidermis was placed in TrypLE (Invitrogen, CA, USA) solution and gently shaken for 20 minutes at 37 °C. The obtained cells were cultured in KC basal medium (Invitrogen) supplemented with 0.06 mM calcium chloride and human keratinocyte growth supplement (Gibco, Grand Island, NY, USA).

### Cell culture and treatment

We purchased the human keratinogenic cell line HaCaT from ATCC (Rockville, MD, USA) and cultured it in DMEM medium (Gibco) containing 10% fetal bovine serum (HyClone, Logan, UT, USA). Subsequently, application of 100 ng/ml murine TNF-α (Peprotech, London, UK) or human TNF-α (Peprotech), 50 nM SM-164 (MedChemExpress, NJ, USA), 20 μM Z-VAD-FMK (MedChemExpress) and different concentrations of RIPK1 inhibitors GSK’2982772 (MedChemExpress) and RIPK3 inhibitor GSK’872 (MedChemExpress) were treated for different times. Besides, recombinant mouse IL-36α was purchased from the R&D system (MN, USA). Later, after different times of treatment with IL-36α in primary keratinocytes, the cells were collected for RT-PCR and protein blotting assays.

### Cell viability assay

Cell viability was assessed using a Cell Counting Kit-8 (Yeasen, Shanghai, China) or a CellTiter-Glo Luminescent Cell Viability Assay (Promega, Madison, Wisconsin, USA), following the manufacturers’ protocols. OD450 (OD570 for calibration) was detected by a microplate reader (Molecular Devices, Sunnyvale, CA, USA) to indicate the cell viability. Fluorescence signals were detected using an enzyme reader (PerkinElmer Envision).

### Immunofluorescence staining

For immunofluorescence staining of skin tissues, paraffin sections were deparaffinized and antigenically repaired using sodium citrate antigen repair solution (Beyotime, Shanghai, China). After treatment with the blocking solution (Beyotime) for 1 h, the sections were treated with RIPK3 (CST, Danvers, MA, USA, 95702), p-MLKL (Abcam, Cambridge, UK, ab196436), S100A8 (Proteintech, Chicago, IL, USA, 15792-1-AP), fluorescein isothiocyanate (FITC)-labeled Gr-1 mAb (BD Biosciences, USA, 553127), and goat anti-IL-36α (R&D, AF2297) at 4 °C overnight. After washing with washing solution (Beyotime), anti-rabbit or anti-goat secondary antibodies coupled with Alexa Fluor^®^ 647 (Abcam, ab150115) or PE (Proteintech, SA00008-2) were added. After incubation for 1 hour at room temperature, cells were washed and restained with DAPI.

HaCaT cells or mouse primary keratinocytes on coverslips were treated as described previously, and after closure was completed, rabbit anti-Occludin (Abcam, ab216327) and Claudin4 (Abcam, ab15104) were added, respectively, and incubated overnight at 4 °C. After washing with wash solution, anti-rabbit secondary antibodies coupled with Alexa Fluor^®^ 647 (Abcam) or PE (Proteintech) were added. All images were taken by Leica TCS SPS CFSMP microscope (Wetzlar, Germany).

### Flow cytometry assay

Skin tissues were placed in RPMI 1640 medium containing 10% FBS, and 1 mg/ml of COLLAGENASE (SIGMA, C5138) was added, incubated at 37 °C for 2.5 hours, and homogenized to obtain the suspension. Subsequently, the suspension was resuspended by filtration and centrifugation sequentially to obtain the single-cell suspension of skin tissue.

Before flow cytometric analysis, single cells were first washed with PBS to remove cell culture medium. Fixable viability dye eFluor^TM^ 780 (eBioscience, San Diego, CA, USA, 65-0865-14) was used to distinguish live and dead cells in the single-cell suspension. Surface markers were labeled with CD16/CD32 monoclonal antibody (Thermo Fisher Scientific, MA, USA, 14-0161-86) to prevent non-specific binding. Subsequently, cells were stained by incubation with antibodies attached to different fluorescent markers. Flow cytometric analysis was carried out using the BD LSRFortessa. The acquired data were then analyzed using FlowJo V10 software (Treestar, Ashland, OR, USA).

### RNA extraction and RT-PCR

Total RNA was extracted from cells and skin tissues using the RNA simple total RNA kit (Tiangen, Beijing, China) and strictly following the instructions. Then cDNA was synthesized with Hifair^®^ AdvanceFast One-step RT-gDNA Digestion SuperMix for qPCR (Yeasen, Shanghai, China). Subsequently, SYBR^®^ Green Master Mix (Low Rox Plus) (Yeasen) and 7500 Fast Real-Time PCR System (Applied Biosystems, Foster City, CA, USA) and gene-specific primers were used for real-time quantitative PCR analysis. RT-PCR primer sequences are shown in Table S2.

### Cytokines detection by ELISA

Quantification of cytokines in mouse serum using TNF-α, IL-6 and IFN-γ enzyme-linked immunosorbent assay (ELISA) kits (BD Pharmingen, San Diego, CA, USA).

### Western blot analysis

Skin tissues and cells were fully lysed in a lysis solution of sodium dodecyl sulfate (Beyotime) supplemented with protease and phosphatase inhibitors (Beyotime). Protein samples were boiled at 100 ℃ for 10 minutes and then electrophoresed in SDS-PAGE gels, and then the proteins were transferred to nitrocellulose membranes (Bio-Rad, Hercules, CA, USA). The membranes were blocked with skimmed milk powder and incubated with the appropriate proportion of diluted antibodies at 4 °C. The following day after incubation with a proportionate dilution of the HRP-conjugated antibody, the proteins were imaged using the ChemiDoc^TM^ MP Imaging System (Bio-Rad). The following antibodies were used for Western blot analysis: anti-RIPK1 (CST, 3493), anti-p-RIPK1 (phospho-Ser166) (CST, 31122), anti-RIPK3 (CST, 95702), anti-p-RIPK3 (phospho-S232) (Abcam, ab195117), anti-MLKL (CST, 37705), anti-p-MLKL (phospho-S345) (Abcam, ab150115), anti-Occludin (Abcam, ab216327), anti-Claudin4 (Abcam, ab15104), anti-Claudin1 (Abcam, ab15098), anti-p-RIPK1 (phospho-Ser166) (CST, 65746), anti-p-RIPK3 (phospho-Ser227) (CST, 93654), anti RIPK3 (CST, 10188), anti-MLKL (Abcam, ab243142), anti-p-MLKL (phospho-S358) (Abcam, ab187091), anti-IL-36 alpha (R&D), anti-MyD88 (CST, 4283), anti-NF-κB (CST, 4764), anti- p-NF-κB (Phospho-Ser536) (CST, 3033), anti-IκBα (CST, 4812) and anti-p-IκBα (Phospho-Ser32/36) (CST, 9246).

### Data analysis

Data analysis was conducted using GraphPad Prism 9.0 software (San Diego, CA, USA). Experimental results are expressed as the means ± SEM. Statistical significance between groups was determined using Student’s *t*-test, one-way or two-way ANOVA, as appropriate. A *P* value of less than 0.05 was considered statistically significant.

## Supplementary information

Supplementary information

Original western blots

## Data Availability

The datasets used and/or analyzed during the current study are available from the corresponding author upon reasonable request. All data are provided in the main text or supplementary materials.

## References

[1] Kamata M, Tada Y. Dendritic Cells and Macrophages in the Pathogenesis of Psoriasis. Front Immunol. 2022;13:941071. 35837394 10.3389/fimmu.2022.941071 PMC9274091

[2] Benhadou F, Mintoff D, Del Marmol V. Psoriasis: Keratinocytes or Immune Cells - Which Is the Trigger?. Dermatology. 2019;235:91–100. 30566935 10.1159/000495291

[3] Bu J, Ding R, Zhou L, Chen X, Shen E. Epidemiology of Psoriasis and Comorbid Diseases: A Narrative Review. Front Immunol. 2022;13:880201. 35757712 10.3389/fimmu.2022.880201 PMC9226890

[4] Chen HL, Lo CH, Huang CC, Lu MP, Hu PY, Chen CS, et al. Galectin-7 downregulation in lesional keratinocytes contributes to enhanced IL-17A signaling and skin pathology in psoriasis. J Clin Invest. 2021;131:e130740. 33055419 10.1172/JCI130740 PMC7773376

[5] Zhou X, Chen Y, Cui L, Shi Y, Guo C. Advances in the pathogenesis of psoriasis: from keratinocyte perspective. Cell Death Dis. 2022;13:81. 35075118 10.1038/s41419-022-04523-3 PMC8786887

[6] Lorscheid S, Müller A, Löffler J, Resch C, Bucher P, Kurschus FC, et al. Keratinocyte-derived IκBζ drives psoriasis and associated systemic inflammation. JCI Insight. 2019;4:e130835. 31622280 10.1172/jci.insight.130835 PMC6948851

[7] Huang T, Chen S, Ding K, Zheng B, Lv W, Wang X, et al. SLC35E1 promotes keratinocyte proliferation in psoriasis by regulating zinc homeostasis. Cell Death Dis. 2023;14:354. 37296095 10.1038/s41419-023-05874-1 PMC10256760

[8] Li H, Li J, Zhang X, Feng C, Fan C, Yang X, et al. DC591017, a phosphodiesterase-4 (PDE4) inhibitor with robust anti-inflammation through regulating PKA-CREB signaling. Biochem Pharm. 2020;177:113958. 32251674 10.1016/j.bcp.2020.113958

[9] Wang A, Bai Y. Dendritic cells: The driver of psoriasis. Journal Dermatol. 2019;47:104–13. 10.1111/1346-8138.15184 31833093

[10] Chiang CC, Cheng WJ, Korinek M, Lin CY, Hwang TL. Neutrophils in Psoriasis. Front Immunol. 2019;10:2376. 31649677 10.3389/fimmu.2019.02376 PMC6794444

[11] Griffiths CEM, Armstrong AW, Gudjonsson JE, Barker JNWN. Psoriasis. Lancet. 2021;397:1301–15. 33812489 10.1016/S0140-6736(20)32549-6

[12] Newton K. RIPK1 and RIPK3: critical regulators of inflammation and cell death. Trends Cell Biol. 2015;25:347–53. 25662614 10.1016/j.tcb.2015.01.001

[13] Weinlich R, Oberst A, Beere HM, Green DR. Necroptosis in development, inflammation and disease. Nature Rev Mol Cell Biol. 2016;18:127–36. 27999438 10.1038/nrm.2016.149

[14] Wu X, Ma Y, Zhao K, Zhang J, Sun Y, Li Y, et al. The structure of a minimum amyloid fibril core formed by necroptosis-mediating RHIM of human RIPK3. Proceedings Natl Acad Sci. 2021;118:e2022933118. 10.1073/pnas.2022933118 PMC8040640 33790016

[15] Seo J, Nam YW, Kim S, Oh D-B, Song J. Necroptosis molecular mechanisms: Recent findings regarding novel necroptosis regulators. Experimental Mol Med. 2021;53:1007–17. 10.1038/s12276-021-00634-7 PMC8166896 34075202

[16] Chen X, Li W, Ren J, Huang D, He W-t, Song Y, et al. Translocation of mixed lineage kinase domain-like protein to plasma membrane leads to necrotic cell death. Cell Res. 2013;24:105–21. 24366341 10.1038/cr.2013.171 PMC3879712

[17] Dondelinger Y, Declercq W, Montessuit S, Roelandt R, Goncalves A, Bruggeman I, et al. MLKL Compromises Plasma Membrane Integrity by Binding to Phosphatidylinositol Phosphates. Cell Rep. 2014;7:971–81. 24813885 10.1016/j.celrep.2014.04.026

[18] Liu X, Xie X, Ren Y, Shao Z, Zhang N, Li L, et al. The role of necroptosis in disease and treatment. MedComm. 2021;2:730–55. 34977874 10.1002/mco2.108 PMC8706757

[19] Schünke H, Göbel U, Dikic I, Pasparakis M. OTULIN inhibits RIPK1-mediated keratinocyte necroptosis to prevent skin inflammation in mice. Nature Commun. 2021;12:e130835. 10.1038/s41467-021-25945-1 PMC8501112 34625557

[20] Lecomte K, Toniolo A, Hoste E. Cell death as an architect of adult skin stem cell niches. Cell Death Differ. 2024;31:957–69. 38649745 10.1038/s41418-024-01297-3 PMC11303411

[21] Duan XR, Liu XX, Liu N, Huang YQ, Jin ZL, Zhang S, et al. Inhibition of keratinocyte necroptosis mediated by RIPK1/RIPK3/MLKL provides a protective effect against psoriatic inflammation. Cell Death Dis. 2020;11:134. 32075957 10.1038/s41419-020-2328-0 PMC7031250

[22] Li J, Liu X, Liu Y, Huang F, Liang J, Lin Y, et al. Saracatinib inhibits necroptosis and ameliorates psoriatic inflammation by targeting MLKL. Cell Death Dis. 2024;15:122. 38331847 10.1038/s41419-024-06514-y PMC10853205

[23] Guo Y, Jin L, Dong L, Zhang M, Kuang Y, Chen X, et al. NHWD-1062 ameliorates inflammation and proliferation by the RIPK1/NF-κB/TLR1 axis in Psoriatic Keratinocytes. Biomedicine Pharmacother. 2023;162:114638. 10.1016/j.biopha.2023.114638 37011486

[24] Weisel K, Berger S, Papp K, Maari C, Krueger JG, Scott N, et al. Response to Inhibition of Receptor-Interacting Protein Kinase 1 (RIPK1) in Active Plaque Psoriasis: A Randomized Placebo-Controlled Study. Clinical Pharmacol Therapeutics. 2020;108:808–16. 10.1002/cpt.1852 PMC7540322 32301501

[25] Ludbrook VJ, Budd DC, Thorn K, Tompson D, Votta BJ, Walker L, et al. Inhibition of Receptor-Interacting Protein Kinase 1 in Chronic Plaque Psoriasis: A Multicenter, Randomized, Double-Blind, Placebo-Controlled Study. Dermatology Ther. 2024;14:489–504. 10.1007/s13555-024-01097-0 PMC10890982 38372938

[26] Dannappel M, Vlantis K, Kumari S, Polykratis A, Kim C, Wachsmuth L, et al. RIPK1 maintains epithelial homeostasis by inhibiting apoptosis and necroptosis. Nature. 2014;513:90–4. 25132550 10.1038/nature13608 PMC4206266

[27] Honda T, Yamamoto O, Sawada Y, Egawa G, Kitoh A, Otsuka A, et al. Receptor-interacting protein kinase 3 controls keratinocyte activation in a necroptosis-independent manner and promotes psoriatic dermatitis in mice. J Allergy Clin Immun. 2017;140:619–22.e6. 28342910 10.1016/j.jaci.2017.02.027

[28] Sachen KL, Arnold Greving CN, Towne JE. Role of IL-36 cytokines in psoriasis and other inflammatory skin conditions. Cytokine. 2022;156:155897. 35679693 10.1016/j.cyto.2022.155897

[29] Johnston A, Xing X, Guzman AM, Riblett M, Loyd CM, Ward NL, et al. IL-1F5, -F6, -F8, and -F9: A Novel IL-1 Family Signaling System That Is Active in Psoriasis and Promotes Keratinocyte Antimicrobial Peptide Expression. Journal Immunol. 2011;186:2613–22. 21242515 10.4049/jimmunol.1003162 PMC3074475

[30] Yuan ZC, Xu WD, Liu XY, Liu XY, Huang AF, Su LC. Biology of IL-36 Signaling and Its Role in Systemic Inflammatory Diseases. Front Immunol. 2019;10:2532. 31736959 10.3389/fimmu.2019.02532 PMC6839525

[31] Su Z, Paulsboe S, Wetter J, Salte K, Kannan A, Mathew S, et al. IL-36 receptor antagonistic antibodies inhibit inflammatory responses in preclinical models of psoriasiform dermatitis. Experimental Dermatol. 2018;28:113–20. 10.1111/exd.13841 30417427

[32] Armstrong AW, Elston CA, Elewski BE, Ferris LK, Gottlieb AB, Lebwohl MG, et al. Generalized pustular psoriasis: A consensus statement from the National Psoriasis Foundation. Journal Am Acad Dermatol. 2023;90:727–30. 37838256 10.1016/j.jaad.2023.09.080

[33] Xu X, Zhang Y, Pan Z, Zhang X, Liu X, Tang L, et al. Genome-wide DNA methylation of Munro’s microabscess reveals the epigenetic regulation in the pathogenesis of psoriasis. Front Immunol. 2022;13:1057839. 36569916 10.3389/fimmu.2022.1057839 PMC9773074

[34] Steffen C. William John Munro and Munro’s abscess, and Franz Kogoj and Kogoj’s spongiform pustule. Am J Dermatopathol. 2002;24:364–8. 12142621 10.1097/00000372-200208000-00016

[35] van der Fits L, Mourits S, Voerman JS, Kant M, Boon L, Laman JD, et al. Imiquimod-induced psoriasis-like skin inflammation in mice is mediated via the IL-23/IL-17 axis. J Immunol. 2009;182:5836–45. 19380832 10.4049/jimmunol.0802999

[36] Aochi S, Tsuji K, Sakaguchi M, Huh N, Tsuda T, Yamanishi K, et al. Markedly elevated serum levels of calcium-binding S100A8/A9 proteins in psoriatic arthritis are due to activated monocytes/macrophages. J Am Acad Dermatol. 2011;64:879–87. 21315480 10.1016/j.jaad.2010.02.049

[37] Schonthaler HB, Guinea-Viniegra J, Wculek SK, Ruppen I, Ximénez-Embún P, Guío-Carrión A, et al. S100A8-S100A9 protein complex mediates psoriasis by regulating the expression of complement factor C3. Immunity. 2013;39:1171–81. 24332034 10.1016/j.immuni.2013.11.011

[38] Bai F, Fan C, Lin X, Wang HY, Wu B, Feng CL, et al. Hemin protects UVB-induced skin damage through inhibiting keratinocytes apoptosis and reducing neutrophil infiltration. J Photochem Photobio B. 2023;238:112604. 10.1016/j.jphotobiol.2022.112604 36525776

[39] Zhao J, Jitkaew S, Cai Z, Choksi S, Li Q, Luo J, et al. Mixed lineage kinase domain-like is a key receptor interacting protein 3 downstream component of TNF-induced necrosis. Proceedings Natl Acad Sci. 2012;109:5322–7. 10.1073/pnas.1200012109 PMC3325682 22421439

[40] Vandenabeele P, Declercq W, Van Herreweghe F, Vanden Berghe T. The Role of the Kinases RIP1 and RIP3 in TNF-Induced Necrosis. Sci Signal. 2010;3:re4. 20354226 10.1126/scisignal.3115re4

[41] Silke J, Rickard JA, Gerlic M. The diverse role of RIP kinases in necroptosis and inflammation. Nat Immunol. 2015;16:689–97. 26086143 10.1038/ni.3206

[42] Furue M, Furue K, Tsuji G, Nakahara T. Interleukin-17A and Keratinocytes in Psoriasis. Int J Mol Sci. 2020;21:1275. 32070069 10.3390/ijms21041275 PMC7072868

[43] Guo J, Zhang H, Lin W, Lu L, Su J, Chen X. Signaling pathways and targeted therapies for psoriasis. Signal Transduct Target Ther. 2023;8:437. 38008779 10.1038/s41392-023-01655-6 PMC10679229

[44] Pasparakis M, Vandenabeele P. Necroptosis and its role in inflammation. Nature. 2015;517:311–20. 25592536 10.1038/nature14191

[45] Zhang H, Hou W, Henrot L, Schnebert S, Dumas M, Heusèle C, et al. Modelling epidermis homoeostasis and psoriasis pathogenesis. J R Soc Interface. 2015;12:20141071. 25566881 10.1098/rsif.2014.1071 PMC4305409

[46] Locksley RM, Killeen N, Lenardo MJ. The TNF and TNF receptor superfamilies: integrating mammalian biology. Cell. 2001;104:487–501. 11239407 10.1016/s0092-8674(01)00237-9

[47] Kabashima K, Honda T, Ginhoux F, Egawa G. The immunological anatomy of the skin. Nat Rev Immunol. 2019;19:19–30. 30429578 10.1038/s41577-018-0084-5

[48] Shou Y, Yang L, Yang Y, Xu J. Inhibition of keratinocyte ferroptosis suppresses psoriatic inflammation. Cell Death Dis. 2021;12:1009. 34707088 10.1038/s41419-021-04284-5 PMC8551323

[49] Jiang BW, Zhang WJ, Wang Y, Tan LP, Bao YL, Song ZB, et al. Convallatoxin induces HaCaT cell necroptosis and ameliorates skin lesions in psoriasis-like mouse models. Biomed Pharmacother. 2020;121:109615. 31707343 10.1016/j.biopha.2019.109615

[50] Lian N, Chen Y, Chen S, Zhang Y, Chen H, Yang Y, et al. Gasdermin D-mediated keratinocyte pyroptosis as a key step in psoriasis pathogenesis. Cell Death Dis. 2023;14:595. 37673869 10.1038/s41419-023-06094-3 PMC10482869

[51] Newton K, Dugger DL, Maltzman A, Greve JM, Hedehus M, Martin-McNulty B, et al. RIPK3 deficiency or catalytically inactive RIPK1 provides greater benefit than MLKL deficiency in mouse models of inflammation and tissue injury. Cell Death Differ. 2016;23:1565–76. 27177019 10.1038/cdd.2016.46 PMC5072432

[52] Bertheloot D, Latz E, Franklin BS. Necroptosis, pyroptosis and apoptosis: an intricate game of cell death. Cellular Mol Immunol. 2021;18:1106–21. 33785842 10.1038/s41423-020-00630-3 PMC8008022

[53] Lawlor KE, Khan N, Mildenhall A, Gerlic M, Croker BA, D’Cruz AA, et al. RIPK3 promotes cell death and NLRP3 inflammasome activation in the absence of MLKL. Nat Commun. 2015;6:6282. 25693118 10.1038/ncomms7282 PMC4346630

[54] Zhan L, Lu X, Xu W, Sun W, Xu E. Inhibition of MLKL-dependent necroptosis via downregulating interleukin-1R1 contributes to neuroprotection of hypoxic preconditioning in transient global cerebral ischemic rats. J Neuroinflammation. 2021;18:97. 33879157 10.1186/s12974-021-02141-y PMC8056617

[55] Bernardo D, Thaçi D, Torres T. Spesolimab for the Treatment of Generalized Pustular Psoriasis. Drugs. 2023;84:45–58. 38114719 10.1007/s40265-023-01988-0 PMC10789831

[56] Ghoreschi K, Balato A, Enerbäck C, Sabat R. Therapeutics targeting the IL-23 and IL-17 pathway in psoriasis. Lancet. 2021;397:754–66. 33515492 10.1016/S0140-6736(21)00184-7

[57] Martin-Sanchez D, Guerrero-Mauvecin J, Fontecha-Barriuso M, Mendez-Barbero N, Saiz ML, Lopez-Diaz AM, et al. Bone Marrow-Derived RIPK3 Mediates Kidney Inflammation in Acute Kidney Injury. J Am Soc Nephrol. 2022;33:357–73. 35046131 10.1681/ASN.2021030383 PMC8819996

[58] Xie Y, Zhao Y, Shi L, Li W, Chen K, Li M, et al. Gut epithelial TSC1/mTOR controls RIPK3-dependent necroptosis in intestinal inflammation and cancer. J Clin Invest. 2020;130:2111–28. 31961824 10.1172/JCI133264 PMC7108921

[59] Qu X, Yang T, Wang X, Xu D, Yu Y, Li J, et al. Macrophage RIPK3 triggers inflammation and cell death via the XBP1-Foxo1 axis in liver ischaemia-reperfusion injury. JHEP Rep. 2023;5:100879. 37841640 10.1016/j.jhepr.2023.100879 PMC10568422

[60] Park H-H, Kim H-R, Park S-Y, Hwang S-M, Hong SM, Park S, et al. RIPK3 activation induces TRIM28 derepression in cancer cells and enhances the anti-tumor microenvironment. Molecular Cancer. 2021;20:107. 34419074 10.1186/s12943-021-01399-3 PMC8379748

[61] Afonso MB, Islam T, Magusto J, Amorim R, Lenoir V, Simoes RF, et al. RIPK3 dampens mitochondrial bioenergetics and lipid droplet dynamics in metabolic liver disease. Hepatology. 2023;77:1319–34. 36029129 10.1002/hep.32756 PMC10026966

[62] Weindel CG, Martinez EL, Zhao X, Mabry CJ, Bell SL, Vail KJ, et al. Mitochondrial ROS promotes susceptibility to infection via gasdermin D-mediated necroptosis. Cell. 2022;185:3214–31.e23. 35907404 10.1016/j.cell.2022.06.038 PMC9531054

[63] Doglio MG, Verboom L, Ruilova Sosoranga E, Frising UC, Asaoka T, Gansemans Y, et al. Myeloid OTULIN deficiency couples RIPK3-dependent cell death to Nlrp3 inflammasome activation and IL-1β secretion. Sci Immunol. 2023;8:eadf4404. 38000038 10.1126/sciimmunol.adf4404

[64] Choi SW, Park HH, Kim S, Chung JM, Noh HJ, Kim SK, et al. PELI1 Selectively Targets Kinase-Active RIP3 for Ubiquitylation-Dependent Proteasomal Degradation. Mol Cell. 2018;70:920–35.e7. 29883609 10.1016/j.molcel.2018.05.016

[65] Elias M, Zhao S, Le HT, Wang J, Neurath MF, Neufert C. et al. IL-36 in chronic inflammation and fibrosis - bridging the gap? J Clin Invest. 2021;131:e144336. 33463541 10.1172/JCI144336 PMC7810483

[66] Oeckinghaus A, Hayden MS, Ghosh S. Crosstalk in NF-κB signaling pathways. Nat Immunol. 2011;12:695–708. 21772278 10.1038/ni.2065

[67] Ding X, Bloch W, Iden S, Rüegg MA, Hall MN, Leptin M, et al. mTORC1 and mTORC2 regulate skin morphogenesis and epidermal barrier formation. Nat Commun. 2016;7:13226. 27807348 10.1038/ncomms13226 PMC5095294

[68] Müller A, Hennig A, Lorscheid S, Grondona P, Schulze-Osthoff K, Hailfinger S, et al. IκBζ is a key transcriptional regulator of IL-36–driven psoriasis-related gene expression in keratinocytes. Proceedings Natl Acad Sci. 2018;115:10088–93. 10.1073/pnas.1801377115 PMC6176600 30224457

[69] Li F, Adase CA, Zhang LJ. Isolation and Culture of Primary Mouse Keratinocytes from Neonatal and Adult Mouse Skin. J Vis Exp. 2017;56027. 10.3791/56027 PMC5612483 28745643

