# Supplementary material for: RIPK3 promotes skin inflammation by enhancing IL-36α signaling and necroptosis in keratinocytes
Source: Cell Death Dis. 2025 Oct 24;16(1):759. doi: 10.1038/s41419-025-08096-9 (PMC12552518; doi:10.1038/s41419-025-08096-9)
Supplement: Supplementary file 1 — Supplementary information [file 41419_2025_8096_MOESM1_ESM.docx]

**Supplementary Information**

**Title:** RIPK3 promotes skin inflammation by enhancing IL-36α signaling and necroptosis in keratinocytes

**Running Title:** RIPK3 promotes skin inflammation via IL-36α and necroptosis

Qing-qing Li^1,2, #^, Tao Yang^2,3, #^, Jin-jin Ren^1,2^, Zhi-zhen Hui^4^, Shu-yue Lei^2,3^, Chun-lan Feng^2^, Xiao-qian Yang^2^ and Wei Tang^1,2,3,4, *^

^1^The Institute of Clinical Pharmacology, Anhui Medical University, Key Laboratory of Anti-inflammatory and Immune Medicine, Ministry of Education, Anhui Collaborative Innovation Center of Anti-inflammatory and Immune Medicine, Hefei 230032, China

^2^State Key Laboratory of Chemical Biology, Shanghai Institute of Materia Medica, Chinese Academy of Sciences, Shanghai 201203, China

^3^School of Pharmacy, University of Chinese Academy of Sciences, Beijing 100049, China

^4^School of Chinese Materia Medica, Nanjing University of Chinese Medicine, Nanjing, 210023, China

**Supplementary Figures 1 to 6**

**Supplementary Tables 1 to 2**


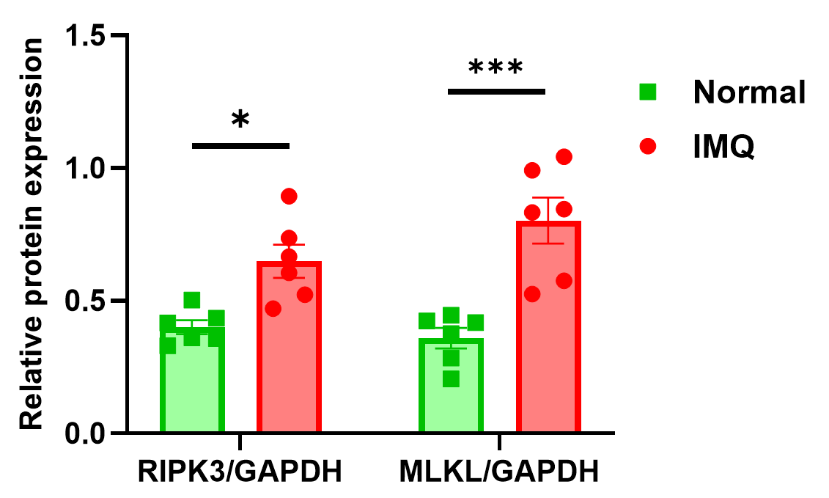


**Supplementary Fig. 1 RIPK3 is upregulated in IMQ-treated mice.** Densitometric analysis of RIPK3 and MLKL protein expression levels in skin tissue of Balb/c mice. All dates are shown as means ± SEM. ^*^*P* < 0.05, ^***^*P* < 0.001, compared as indicated, were measured by Student’s *t*-test.


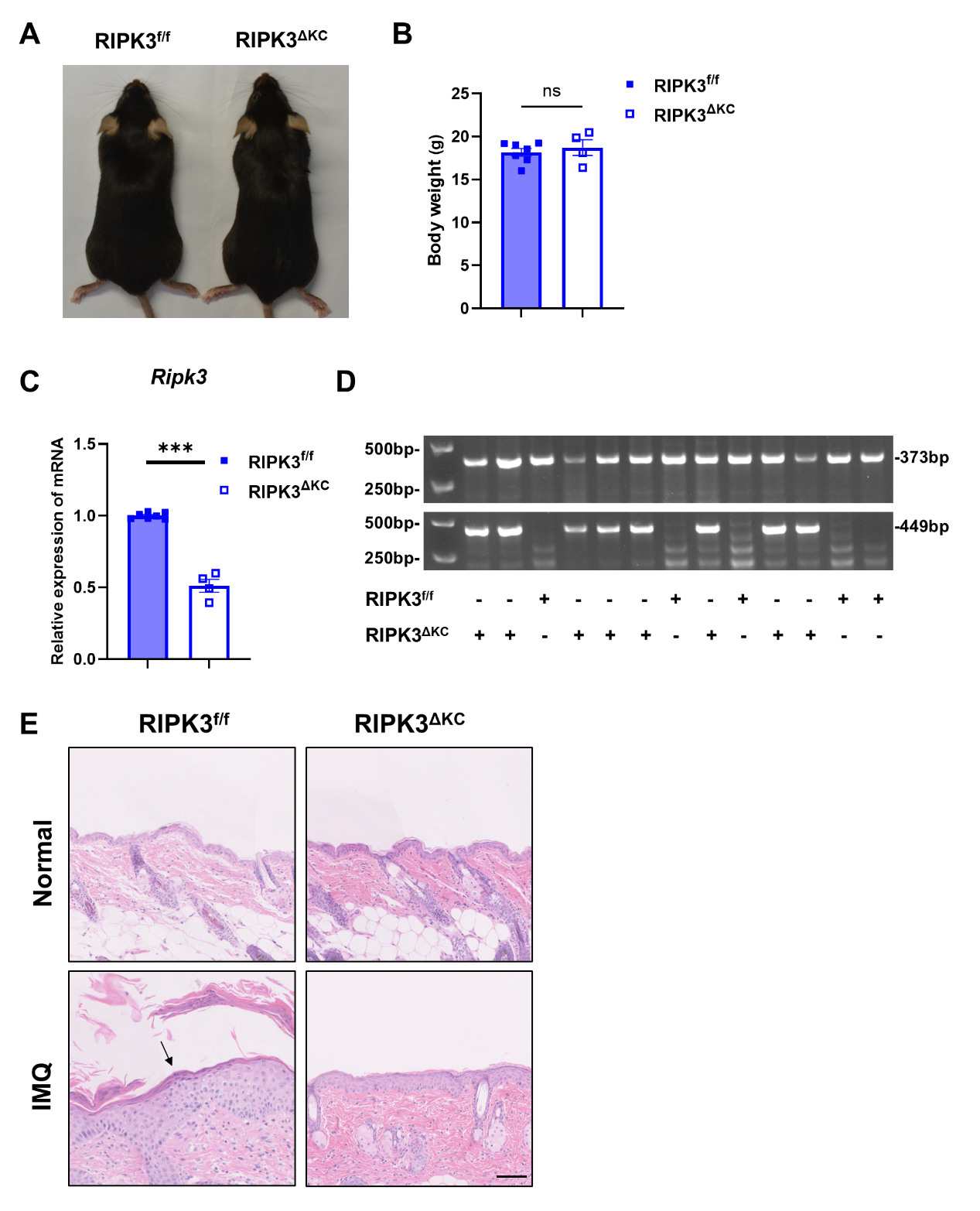


**Supplementary Fig. 2 RIPK3^ΔKC^ attenuates the severity of IMQ-induced skin damage. A** Photographs of mice with the indicated genotypes at the age of 6-8 weeks. **B** Body weight measurements of RIPK3^f/f^ and RIPK3^ΔKC^ mice at 6-8 weeks of age. **C** RT-PCR analysis of RIPK3 mRNA expression in skin from RIPK3^f/f^ and RIPK3^ΔKC^ mice. RIPK3^f/f^ (n = 7), RIPK3^ΔKC^ (n = 4). **D** Typical PCR results for genomic identification of RIPK3^f/f^ and RIPK3^ΔKC^ mice. RIPK3^f/f^ allele yields a 373 bp PCR product, while the Cre transgene produces a 449 bp amplicon. **E** Representative H&E staining images in each group. Scale bars, 50 μm. All dates are shown as means ± SEM. ^***^*P* < 0.001, ns indicates no significance, compared with RIPK3^f/f^ group, was measured by Student’s *t*-test.


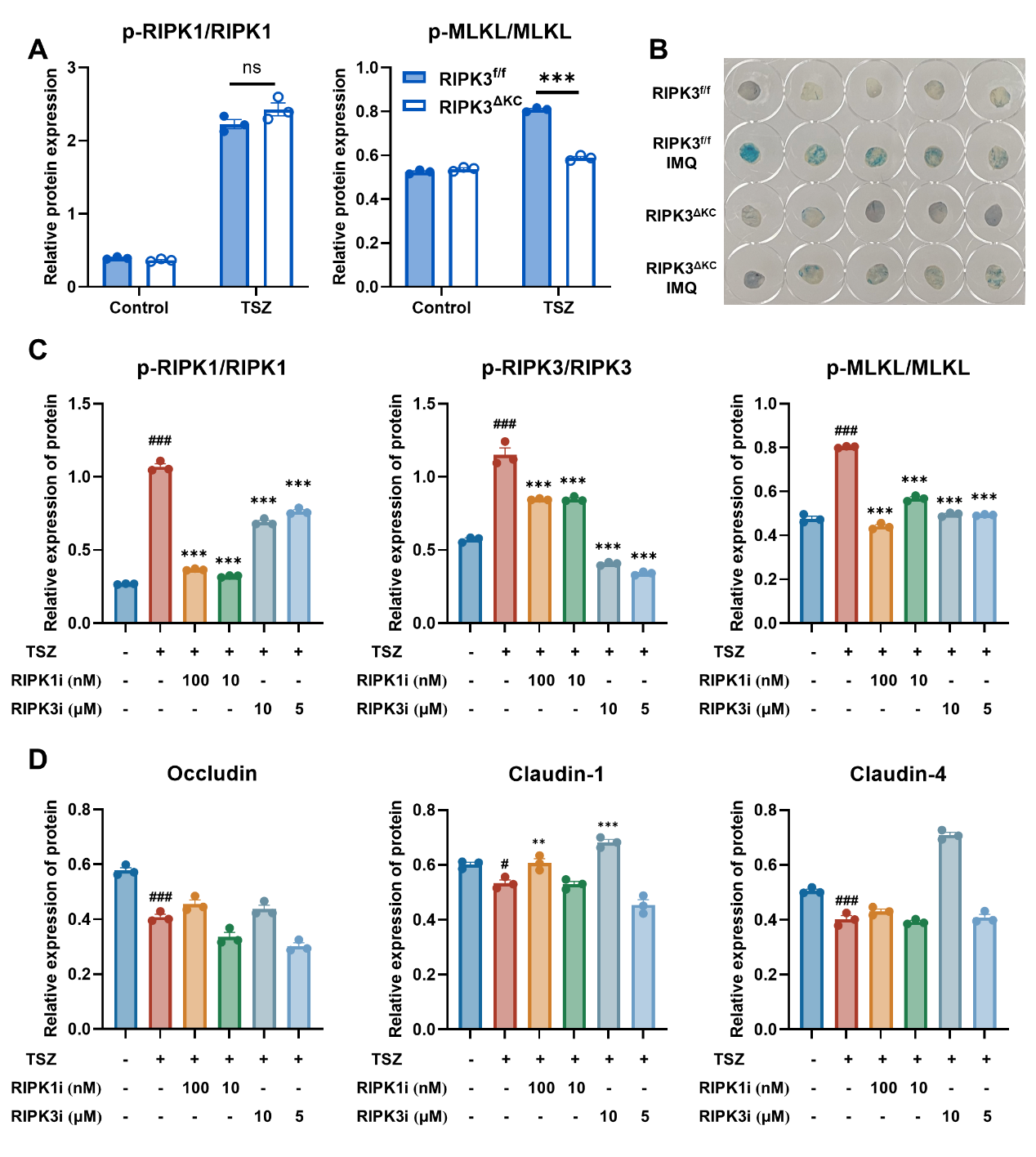
**Supplementary Fig. 3** **Targeting RIPK3 suppresses TSZ-induced keratinocyte necroptosis and inflammation. A** Representative statistical analysis of necroptosis protein expression in primary keratinocytes. **B** Assessment of epidermal barrier integrity in RIPK3^ΔKC^ mice with skin inflammation using toluidine blue staining. **C** Representative statistical analysis of necroptosis protein expression in HaCaT cells. **D** Representative statistical analysis of tight junction protein expression in HaCaT cells. These results are representative of three independent experiments. All dates are shown as means ± SEM. ^**^*P* < 0.01, ^***^*P* < 0.001, ns indicates no significance, compared as indicated, ^#^*P* < 0.05, ^###^*P* < 0.001, compared with control, were measured by one-way or two-way ANOVA. There was a significant difference between the RIPK3^f/f^ and RIPK3^f/f^ TSZ groups.


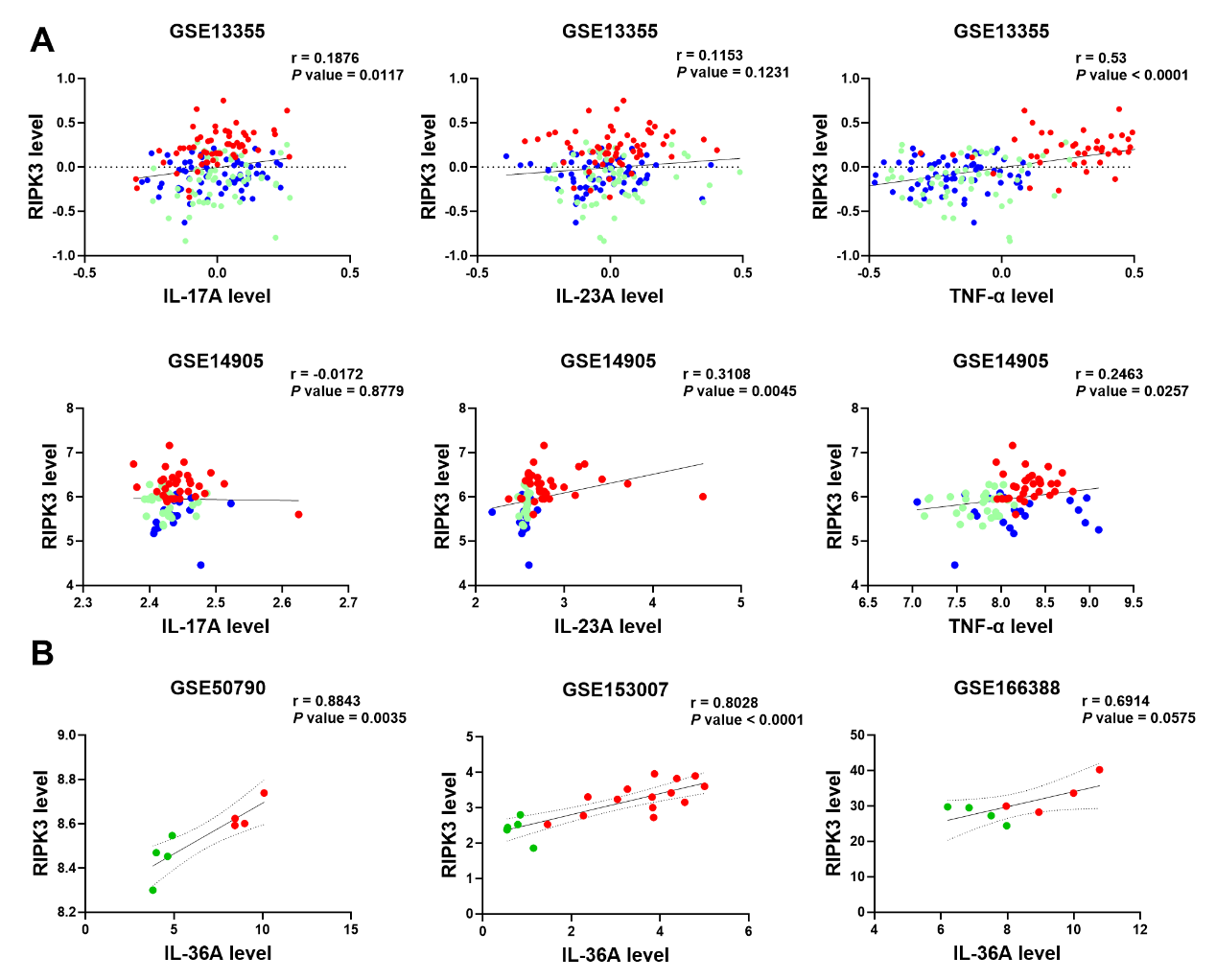
**Supplementary Fig. 4 Correlation between RIPK3 and certain cytokines in patients with psoriasis. A** Correlation of RIPK3 and IL-17A, IL-23A, and TNF-α expression in psoriasis patients in the GEO database. GSE13305: normal (n = 64, green), non-lesional skin (n = 58, blue) and lesional skin (n = 58, red); GSE14905: normal (n = 21, green), non-lesional skin (n = 28, blue) and lesional skin (n = 33, red). **B** Correlation between IL-36α and RIPK3 expression in psoriasis patients in the GEO database. GSE50790: normal (n = 4, green) and lesional skin (n = 4, red); GSE153007: normal (n = 5, green) and lesional skin (n = 14, red); GSE166388: normal (n = 4, green) and lesional skin (n = 4, red).


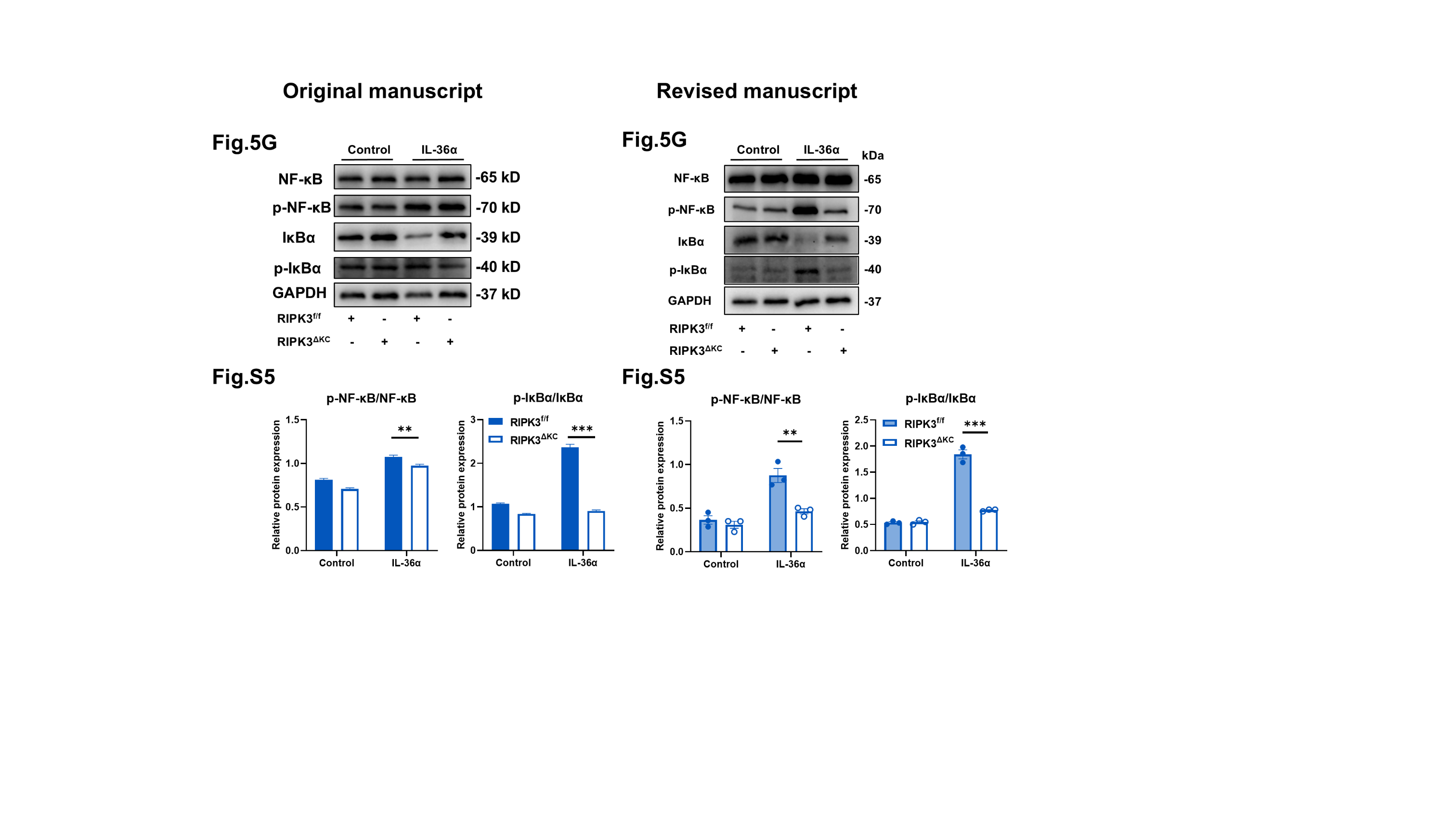


**Supplementary Fig. 5 RIPK3 deficiency reduces the expression of p-NF-κB and p-IκBα in keratinocytes.** Representative statistical analysis of the expression of p-NF-κB and p-IκBα in primary keratinocytes from RIPK3^f/f^ and RIPK3^ΔKC^ mice treated with IL-36α for 15 minutes. These results are representative of three independent experiments. All dates are shown as means ± SEM, ^**^*P* < 0.01, ^***^*P* < 0.001 compared with RIPK3^f/f^ group, were measured by two-way ANOVA. There was a significant difference between the RIPK3^f/f^ and RIPK3^f/f^ TSZ groups.


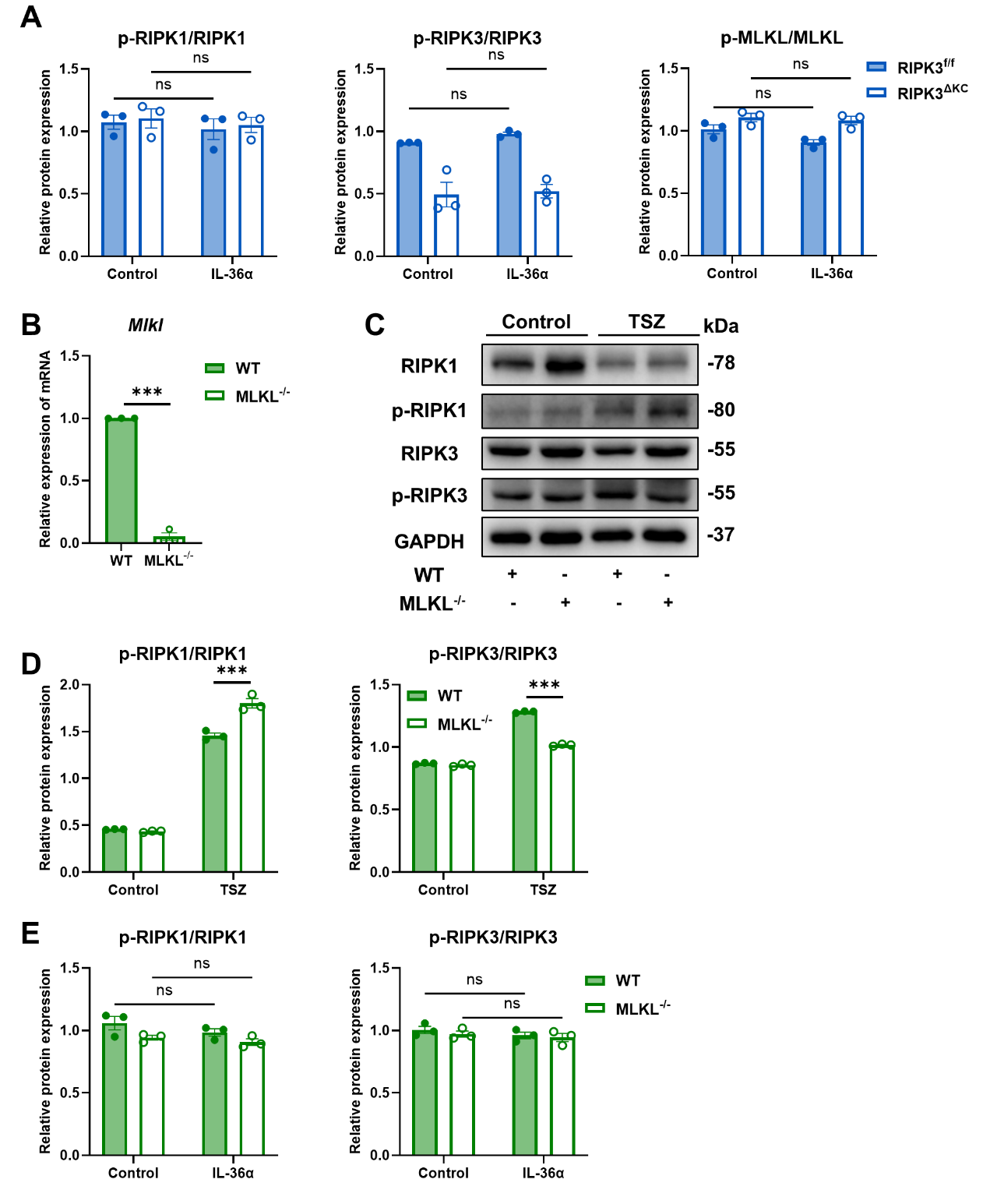


**Supplementary Fig. 6 MLKL-independent modulation of IL-36α signaling by RIPK3 in keratinocytes.** **A** Representative statistical analysis of the necroptosis pathway in primary keratinocytes from RIPK3^f/f^ and RIPK3^ΔKC^ mice treated with IL-36α for 6 hours. **B** RT-PCR detection of mRNA expression levels of MLKL in primary keratinocytes from WT and MLKL^-/-^ mice. Representative western blot (**C**) and statistical analysis (**D**) of the necroptosis pathway in primary keratinocytes from WT and MLKL^-/-^ mice treated with TSZ for 6 hours. **E** Representative statistical analysis of the necroptosis pathway in primary keratinocytes from WT and MLKL^-/-^ mice treated with IL-36α for 6 hours. These results are representative of three independent experiments. All dates are shown as means ± SEM. ^***^*P* < 0.001, compared as indicated, ns indicates no significance, were measured by Student’s *t*-test or two-way ANOVA. Significant differences were observed between the WT and WT TSZ groups.

**Supplementary Table 1** Genotyping Primers

| Gene | | Sequence 5′–3′ |  | |
| --- | --- | --- | --- | --- |
|  | | Forward | Reverse | |
| Ripk3 | GCTACCTACACAGCTTGAACC | | | GTCATTGAGAACTTAGCAGGAG |
| K14 | CGATGGGAAAGTGTAGCCTGCA | | | TCCAGGTATGCTCAGAAAACGCC |
| Mlkl | CATCTCTTTCAGCTATGGATAAATT | | | GCTGGCATTGTTTCCGGCAGTA |

**Supplementary Table 2** Sequences of primers for real-time quantitative PCR.

| Gene | Sequence 5′–3′ |  |
| --- | --- | --- |
|  | Forward | Reverse |
| *β*-Actin | GTGACGTTGACATCCGTAAAGA | GCCGGACTCATCGTACTCC |
| *Gapdh* | AGGTCGGTGTGAACGGATTTG | GGGGTCGTTGATGGCAACA |
| *Ripk3* | GGCACCCTAGCGTACTTGG | GCTGTAGACATCACTCGCTTT |
| *Mlkl* | TTAGGCCAGCTCATCTATGAACA | TGCACACGGTTTCCTAGACG |
| *S100a8* | AAATCACCATGCCCTCTACAAG | CCCACTTTTATCACCATCGCAA |
| *S100a9* | ATACTCTAGGAAGGAAGGACACC | TCCATGATGTCATTTATGAGGGC |
| *Cxcl1* | ACTGCACCCAAACCGAAGTC | TGGGGACACCTTTTAGCATCTT |
| *Il-1β* | GAAATGCCACCTTTTGACAGTG | TGGATGCTCTCATCAGGACAG |
| *Il-17a* | TCAGCGTGTCCAAACACTGAG | CGCCAAGGGAGTTAAAGACTT |
| *Il-17f* | CGTGAAACAGCCATGGTCAA | TTTGGGGTTCTTCCGAGCTG |
| *Tnf-α* | CAGGCGGTGCCTATGTCTC | CGATCACCCCGAAGTTCAGTAG |
| *Ifn-γ* | GCCACGGCACAGTCATTGA | TGCTGATGGCCTGATTGTCTT |
| *Lcn2* | TGGCCCTGAGTGTCATGTG | CTCTTGTAGCTCATAGATGGTGC |
| *S100a7* | TGCTCTTGGATAGTGTGCCTC | GCTCTGTGATGTAGTATGGCTG |
| *Il-1α* | TCTATGATGCAAGCTATGGCTCA | CGGCTCTCCTTGAAGGTGA |
| *Cxcl10* | CCAAGTGCTGCCGTCATTTTC | GGCTCGCAGGGATGATTTCAA |
| *Ccl20* | ACTGTTGCCTCTCGTACATACA | GAGGAGGTTCACAGCCCTTTT |
| *Il-36α* | GCAGCATCACCTTCGCTTAGA | CAGATATTGGCATGGGAGCAAG |
| *Il-36γ* | GTCAGCGTGACTATCCTCCC | TGGCTTCATTGGCTCAGGG |
| IL-1α | TGGTAGTAGCAACCAACGGGA | ACTTTGATTGAGGGCGTCATTC |
| IL-1β | TTCGACACATGGGATAACGAGG | TTTTTGCTGTGAGTCCCGGAG |
| CXCL8 | TTTTGCCAAGGAGTGCTAAAGA | AACCCTCTGCACCCAGTTTTC |
| GAPDH | GGAGCGAGATCCCTCCAAAAT | GGCTGTTGTCATACTTCTCATGG |
