# Supplementary material for: RIPK3 promotes skin inflammation by enhancing IL-36α signaling and necroptosis in keratinocytes
Source: Cell Death Dis. 2025 Oct 24;16(1):759. doi: 10.1038/s41419-025-08096-9 (PMC12552518; doi:10.1038/s41419-025-08096-9)

**Original Western Blot**

**Title:** RIPK3 promotes skin inflammation by enhancing IL-36α signaling and necroptosis in keratinocytes

**Running Title:** RIPK3 promotes skin inflammation via IL-36α and necroptosis

Qing-qing Li^1,2, #^, Tao Yang^2,3, #^, Jin-jin Ren^1,2^, Zhi-zhen Hui^4^, Shu-yue Lei^2,3^, Chun-lan Feng^2^, Xiao-qian Yang^2^ and Wei Tang^1,2,3,4, *^

^1^The Institute of Clinical Pharmacology, Anhui Medical University, Key Laboratory of Anti-inflammatory and Immune Medicine, Ministry of Education, Anhui Collaborative Innovation Center of Anti-inflammatory and Immune Medicine, Hefei 230032, China

^2^State Key Laboratory of Chemical Biology, Shanghai Institute of Materia Medica, Chinese Academy of Sciences, Shanghai 201203, China

^3^School of Pharmacy, University of Chinese Academy of Sciences, Beijing 100049, China

^4^School of Chinese Materia Medica, Nanjing University of Chinese Medicine, Nanjing, 210023, China

**Original Western Blot**


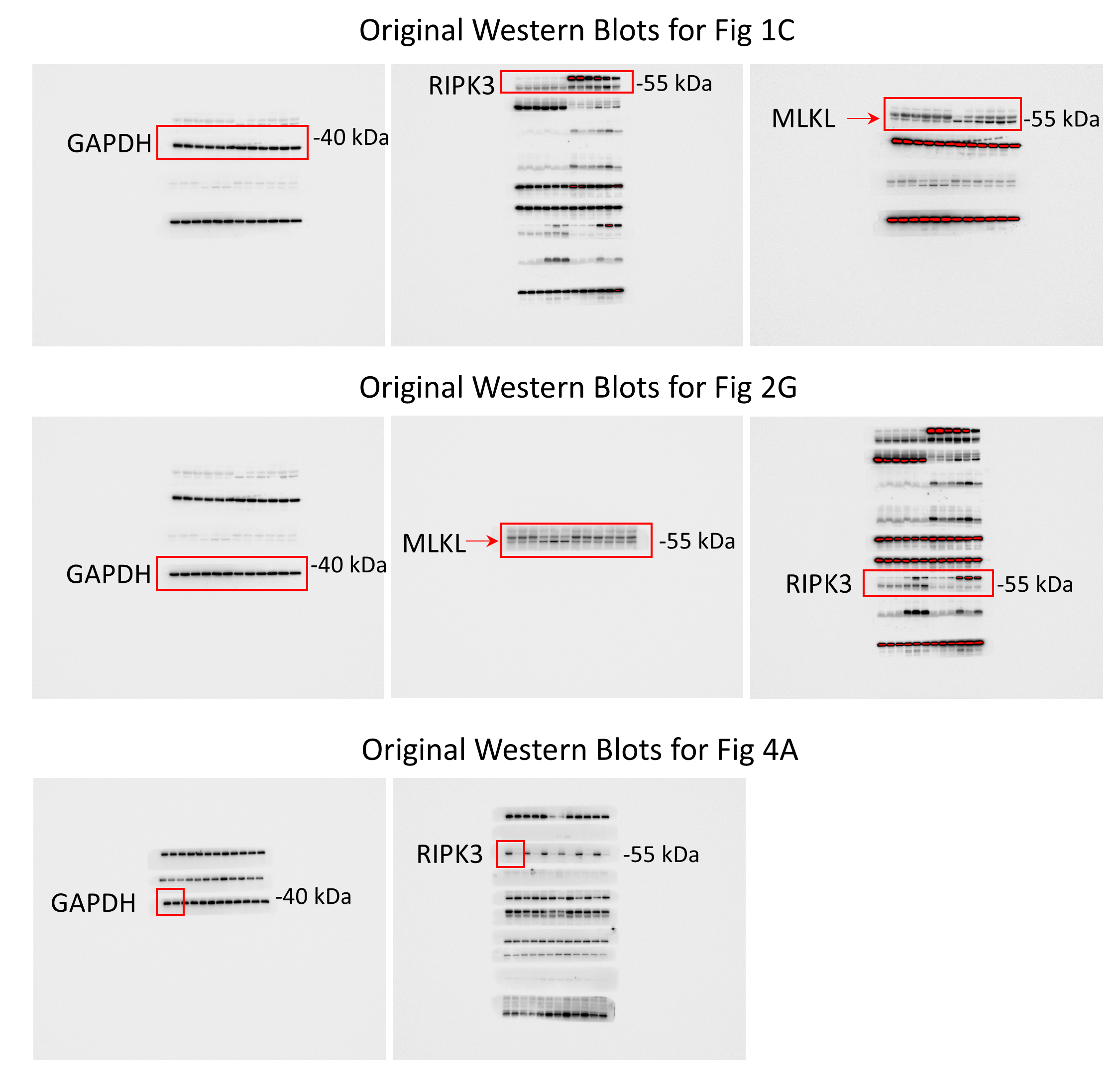


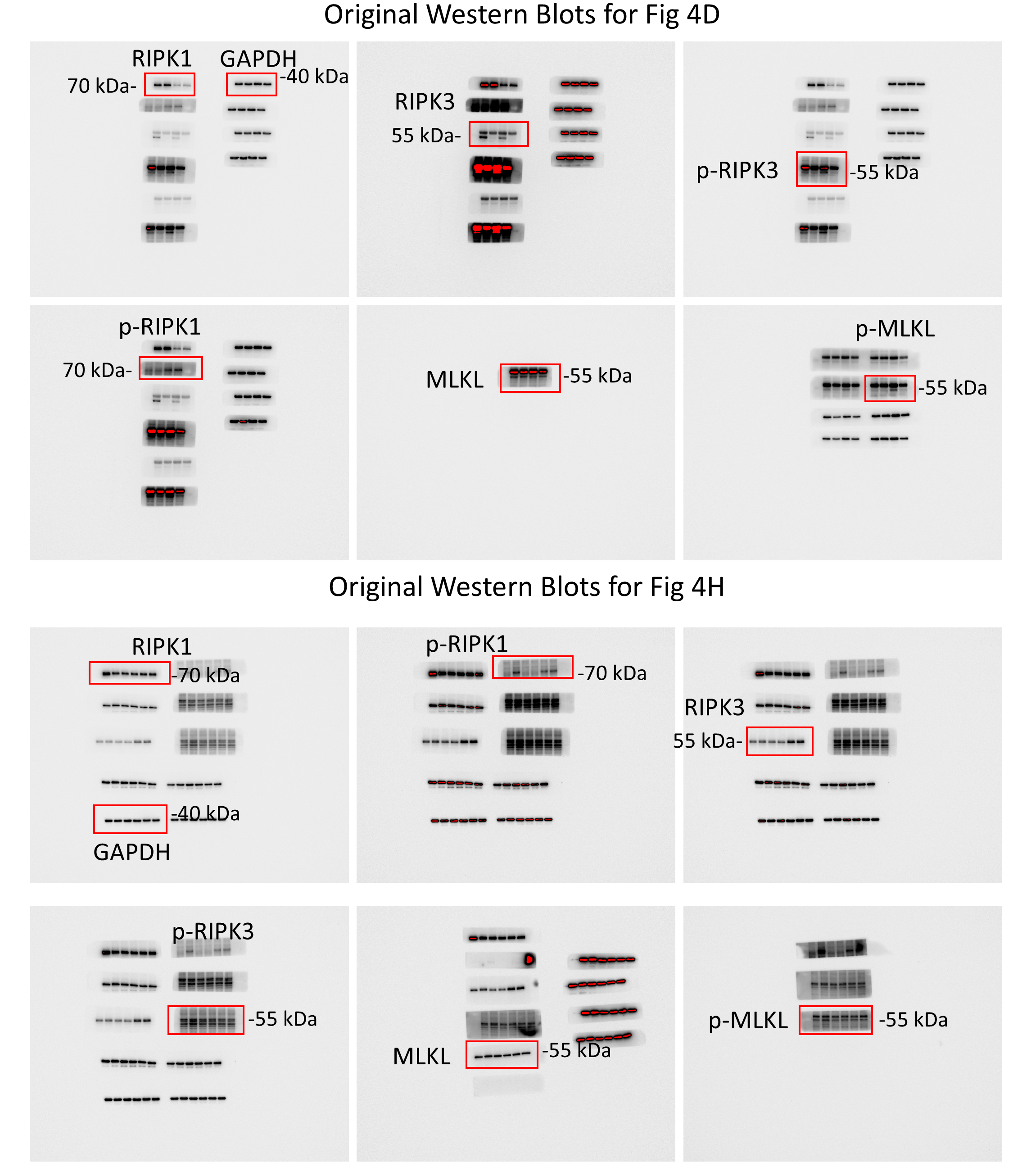


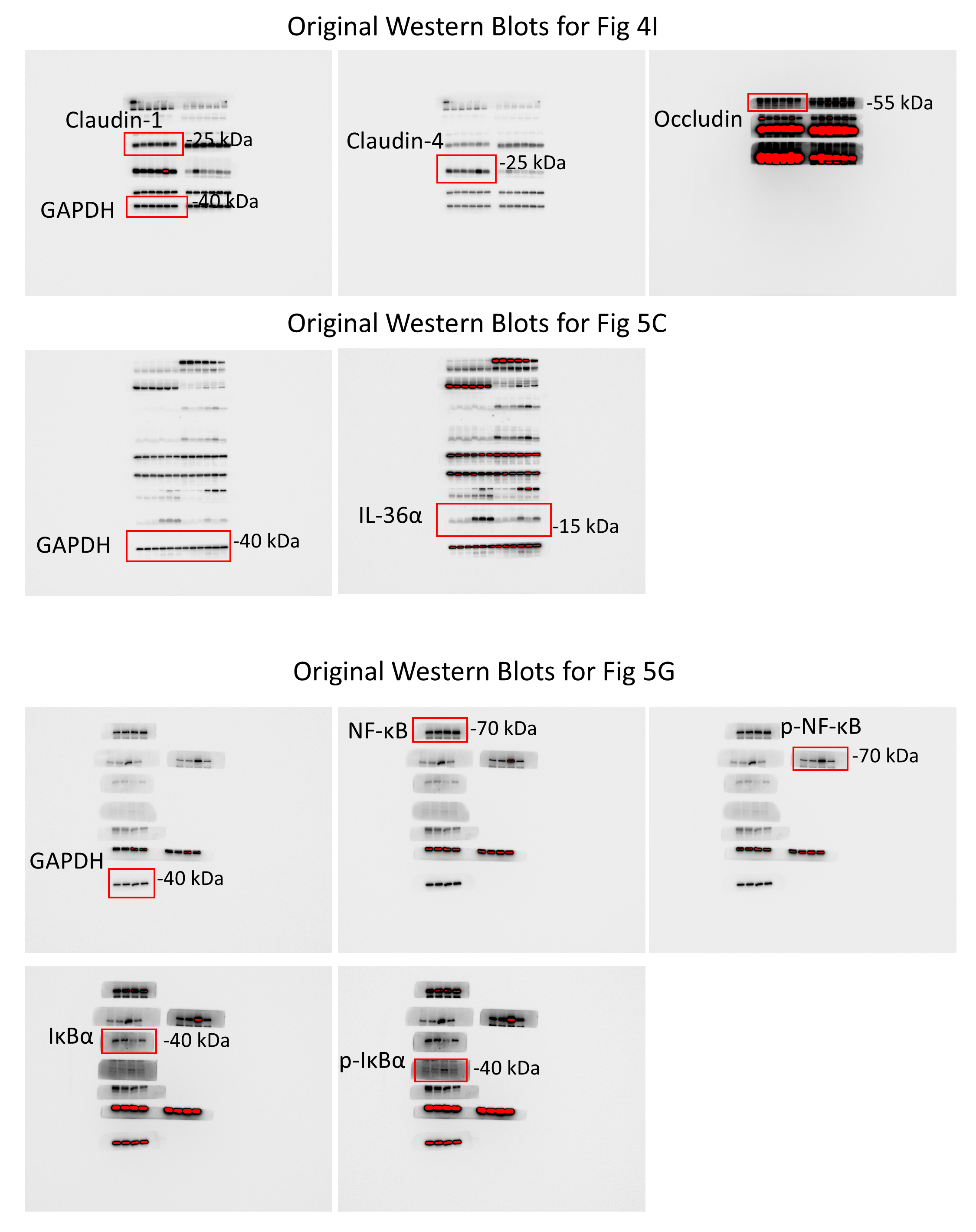

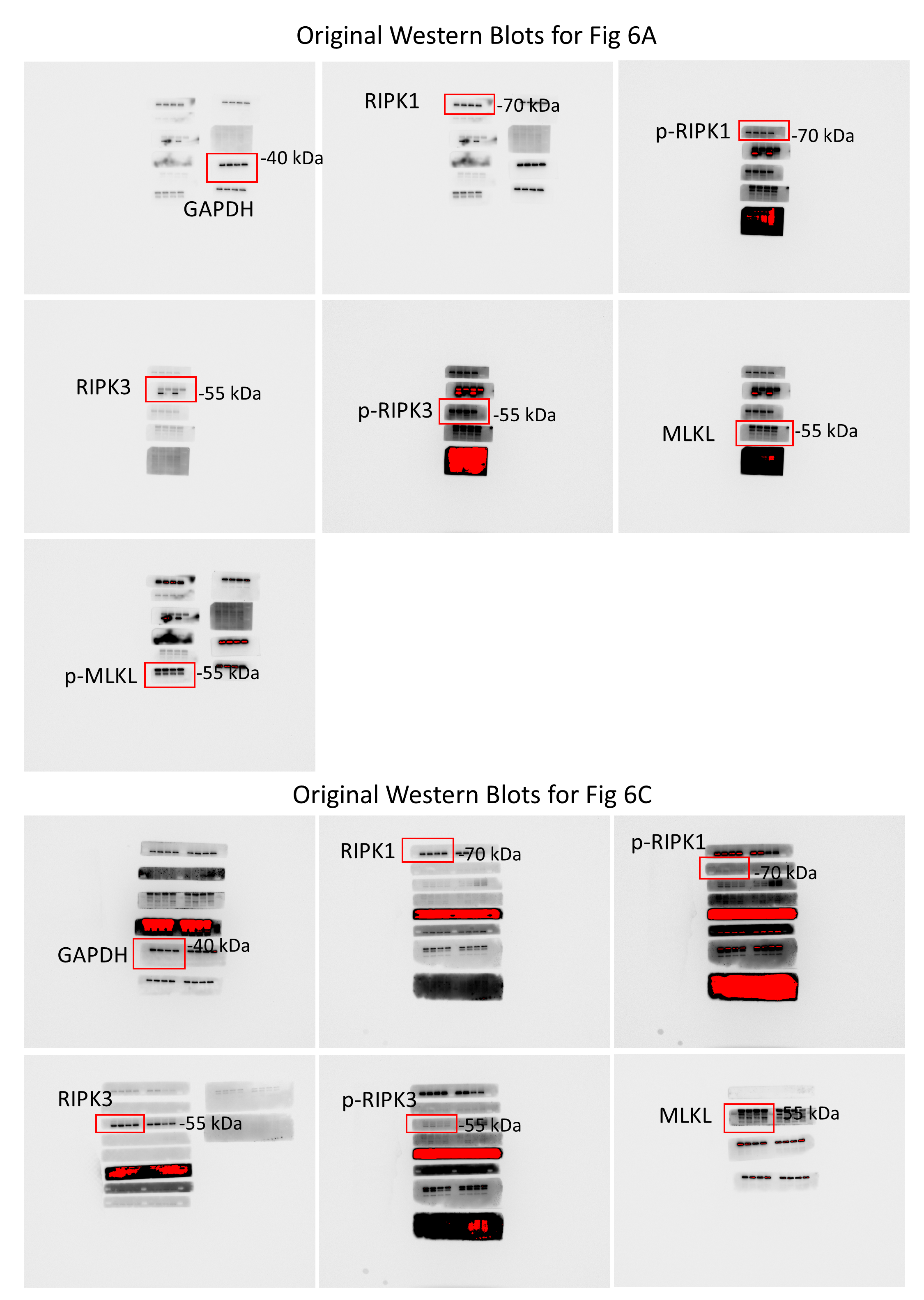

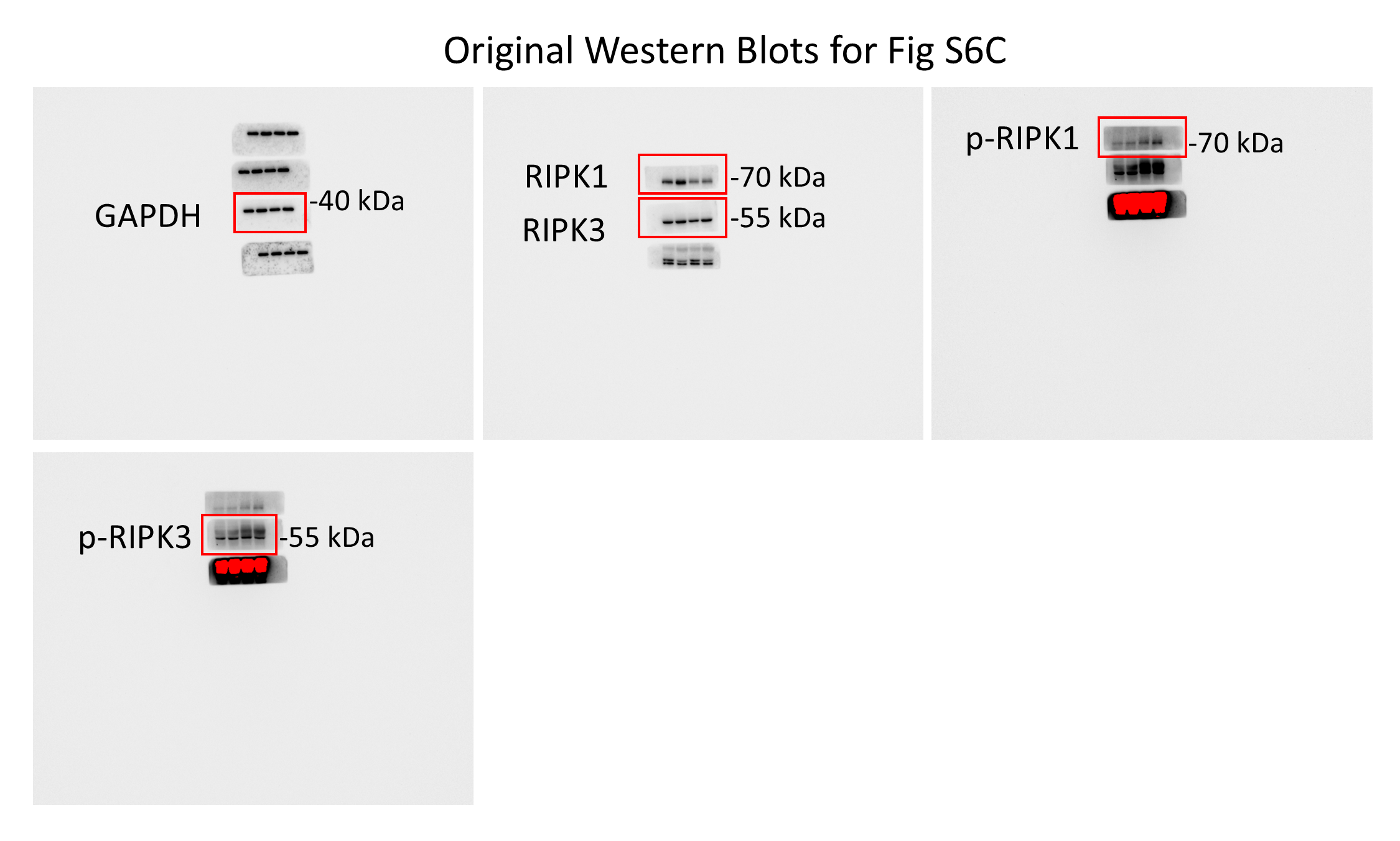

Supplement: Supplementary file 2 — Original western blots [file 41419_2025_8096_MOESM2_ESM.docx]
